# Neurovascular coupling on trial: How the number of trials completed impacts the accuracy and precision of temporally derived neurovascular coupling estimates

**DOI:** 10.1177/0271678X221084400

**Published:** 2022-02-25

**Authors:** Joel S Burma, Rowan K Van Roessel, Ibukunoluwa K Oni, Jeff F Dunn, Jonathan D Smirl

**Affiliations:** 1 Cerebrovascular Concussion Lab, Faculty of Kinesiology, University of Calgary, Alberta, Canada; 2 Sport Injury Prevention Research Centre, Faculty of Kinesiology, University of Calgary, Calgary, Alberta, Canada; 3 Human Performance Laboratory, Faculty of Kinesiology, University of Calgary, Calgary, Alberta, Canada; 4 Libin Cardiovascular Institute of Alberta, University of Calgary, Alberta, Canada; 5 Alberta Children’s Hospital Research Institute, University of Calgary, Calgary, Alberta, Canada; 6 Hotchkiss Brain Institute, University of Calgary, Calgary, Alberta, Canada; 7 Integrated Concussion Research Program, University of Calgary, Calgary, AB, Canada; 8 Department of Clinical Neurosciences, Cumming School of Medicine, University of Calgary, Calgary, Alberta, Canada; 9 Department of Radiology, Cumming School of Medicine, University of Calgary, Calgary, Alberta, Canada

**Keywords:** Neurovascular coupling, transcranial doppler ultrasound, posterior cerebral artery, validity, Where’s Waldo?

## Abstract

Standard practices for quantifying neurovascular coupling (NVC) with transcranial Doppler ultrasound (TCD) require participants to complete one-to-ten repetitive trials. However, limited empirical evidence exists regarding how the number of trials completed influences the validity and reliability of temporally derived NVC metrics. Secondary analyses was performed on 60 young healthy participants (30 females/30 males) who completed eight cyclical eyes-closed (20-seconds), eyes-open (40-seconds) NVC trials, using the *“Where’s Waldo?”* visual paradigm. TCD data was obtained in posterior and middle cerebral arteries (PCA and MCA, respectively). The within-day (n = 11) and between-day (*n* = 17) reliability were assessed at seven- and three-time points, respectively. Repeat testing from the reliability aims were also used for the concurrent validity analysis (*n* = 160). PCA metrics (i.e., baseline, peak, percent increase, and area-under-the-curve) demonstrated five trials produced excellent intraclass correlation coefficient (ICC) 95% confidence intervals for validity and within-day reliability (>0.900), whereas between-day reliability was good-to-excellent (>0.750). Likewise, 95% confidence intervals for coefficient of variation (CoV) measures ranged from acceptable (<20%) to excellent (<5%) with five-or-more trials. Employing fewer than five trials produced poor/unacceptable ICC and CoV metrics. Future NVC, TCD-based research should therefore have participants complete a minimum of five trials when quantifying the NVC response with TCD via a “*Where’s Waldo?*” paradigm.

## Introduction

As the brain has very limited substrate storage, cerebral blood flow (CBF) must be meticulously regulated to ensure a steady supply of oxygen and nutrients are delivered in order to maintain normal functioning and consciousness.^1^ While numerous processes are known to impact cerebral blood flow,^1^ the concept that there is a link between neuronal activity and CBF is known as neurovascular coupling (NVC).^2^ This describes how neural activity and CBF responses are temporally and regionally connected; active (inactive) areas of the brain receive an increased (decreased) supply of nutrients via concurrent alteration in regional blood flow.^2^ NVC has shown to be a sensitive measure of vascular function, and so is increasingly used to study vascular regulation in health and disease.^3^

The NVC response has demonstrated clinical utility in delineating the physiological underpinnings of numerous conditions (e.g., stroke,^4^ pulmonary hypertension,^5^ multiple sclerosis,^6^ sport-related concussion,^7^ etc.). However, if erroneous or unsound methodological approaches are utilized, various studies finding differences between populations may be attributable to measurement error, opposed to true pathophysiological differences. This may produce paradoxical results and confound the literature with lower-quality research. It is therefore imperative consistent and robust methodological approaches capable of producing reliable outputs are employed across research studies within the broader literature.

Temporally derived NVC responses have been widely quantified with transcranial Doppler ultrasound (TCD), which measures cerebral blood velocity (CBV) within the main conduit vessels in the brain.^8^ Nevertheless, a well-established limitation of TCD is its inability to quantify vessel diameter (i.e., poor spatial resolution), thus relying upon an assumption that diameter does not change.^9^ According to Poiseuille’s law, vessel radius will impact total flow to the fourth exponent.^10^ For example, a ∼5–10% increase in diameter will lead to a ∼20–40% increase in CBF.^9^ Despite this consideration, obtaining NVC, autoregulatory, or other cerebrovascular estimates via TCD have demonstrated to produce highly valid and reproducible estimates.^11–15^ More so, despite not being capable to measure diameter, TCD has demonstrated clinical utility to delineate physiological differences compared to healthy controls.^16–18^ An advantageous benefit of TCD is the ability to collect robust data during dynamic movements and maximal exercise,^13–15,19–22^ which is not possible with other neuroimaging equipment (e.g., functional magnetic resonance imaging). Conclusively, in light of these limitations, TCD appears useful under the assumption that vessel diameter does not change.^9^

A review by Stroobant and Vingerhoets^23^ detailed the NVC response has been previously quantified using a wide variety of methodological approaches via different types of auditory, visual, or motor stimuli. Further, a subsequent review by Phillips et al.,^2^ recommended to obtain robust NVC outcome metrics, researchers should have participants complete five-to-ten trials of an active task for at least 30 seconds followed by rest periods.^2^ Despite this recommendation, it is unknown how many trials are required to produce a NVC response that is both highly valid and highly reliable. This may also be dependent upon the methodological approaches utilized, as some techniques produce more robust vascular responses.^24,25^ For example, Smirl et al.,^25^ and Burma et al.,^24^ demonstrated that a complex visual paradigm (“*Where’s Waldo?*”) consistently elicited the most robust response within the main conduit vessel supplying the primary visual cortices (posterior cerebral artery [PCA]) compared to other tasks (e.g., simple shapes or reading). These authors relied upon using eight trials; however, per previous recommendations, it is unknown how the number of trials completed impacts the reliability and validity of these measures.

It is imperative the literature contains empirical evidence regarding the number of trials required for NVC assessments that produce a robust and reliable response. Utilizing an insufficient number of trials may attenuate the ability to understand the pathogenesis of various diseases and disorders, as there could be a limited ability to accurately detect differences compared to either pre-event baselines or control/comparison populations. Finally, to avoid overburdening participants during testing sessions, it is important to employ an appropriate number of trials. The purpose of this paper is to identify the number of trials needed to maximize the signal-to-noise ratio, which will help guide future studies to delineate differences between populations and/or time points within an investigation. To achieve this goal, we sought to determine how the number of repeated activation trials impact the validity, within-day reliability, and between-day reliability of NVC metrics derived using the “*Where’s Waldo?*” visual paradigm.^25^ Based upon previous guidelines,^2^ it was hypothesized valid and reliable NVC estimates can be drawn from five trials; however, the completion of additional successive trials would improve the validity and reliability.

## Material and methods

### Ethical approval

**Figure 1.**
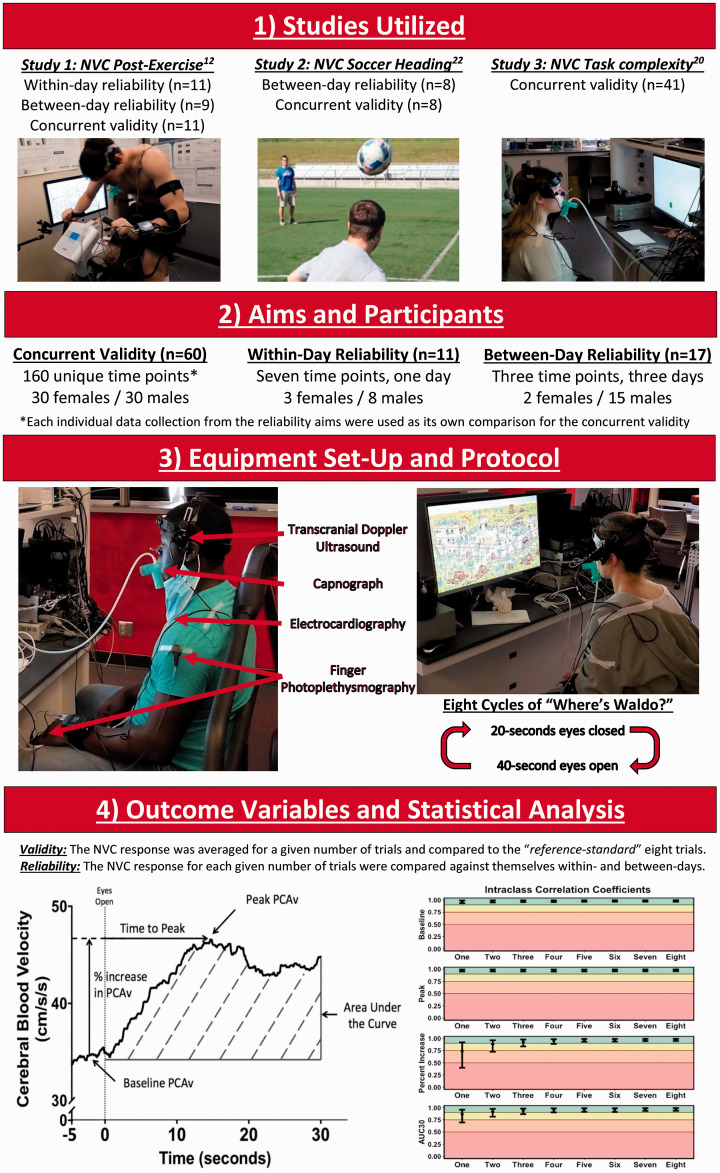
A flow chart delineating the methodological approach of the current investigation. It is important to note baseline data were utilized from all studies and therefore the data were not confounded by the various interventions from the previous studies (i.e., exercise and soccer heading).

Data utilized in this investigation had ethical approval from the Conjoint Health Research Ethics Board at the University of Calgary (REB20-1662 and REB20-2112) and the University of British Columbia clinical ethics review board (H16-00506 and H14-00368). Further, all experimental protocols were conducted in accordance with the guidelines put forth in the Declaration of Helsinki (revised version 2008). Prior to participation, all protocols were thoroughly detailed according to institutional guidelines, all questions were answered, and written informed consent was obtained from all participants prior to the commencement of the study. An illustration of the methodological study design can be seen in Figure 1.

### Participants and study design

Data were utilized and reanalyzed from three previously published investigations,^14,24,26^ which included NVC assessments in young healthy participants. All participants were free of any cardiovascular, neurological, cerebrovascular, musculoskeletal, and respiratory complications. Participants with a previous history of concussion were greater than six months past the date of the injury to ensure this did not confound the data, as NVC recovery has shown to occur approximately one month following the injury.^7^ Theoretically, having the cerebrovasculature free of any disease status would likely produce a robust and consistent NVC response, compared to a clinical population where there could be more variability/inconsistency regarding neuronal signalling.^27^ Therefore, based upon the described research questions, it would be expected the least number of trials required to produce robust NVC results would be within a healthy population.

The first of the three studies examined the extent and duration in which exercise and diurnal variation impacted cerebrovascular,^12–14,20^ cardiovascular,^28^ and oculomotor parameters,^29^ as well as the within- and between-day reliability of the aforementioned metrics. Data were collected November 2016 to August 2018.^14^ These measures were collected on three separate days across seven different time points (i.e., 08:00, 09:30, 10:30, 11:30, 13:30, 15:30, and 17:30), where one of three randomized interventions occurred at 09:00: 1) resting control, 2) moderate-intensity continuous exercise, or 3) high-intensity interval exercise. From this investigation, data were used from the control conditions and from the baseline time points before the moderate- and high-intensity exercise. Therefore, seven time points within a single day were used for the within-day reliability analysis (*n* = 11), whereas the time points at 08:00 across the three days were used for the between-day reliability analysis (*n* = 9). The second study examined how an acute bout of soccer heading altered cerebrovascular function,^26^ cardiovascular parameters, balance assessments, motor function, and blood biomarkers.^30^ These data were similarly collected on three separate days at 08:00, which was used for the between-day analysis (*n* = 8). Data from this heading study were collected from January 2015 to May 2015.^26^ Finally, the third study examined how stimulus duration impacts the NVC coupling response while considering biological sex and self-reported levels of task engagement (*n* = 41). Data for this study were collected at one singular time point from March 2021 to July 2021.^24^ This collection occurred at any time across the typical working day (08:00 – 18:00), as diurnal variation has shown to have minimal impact on NVC metrics.^14^

From the accumulation of studies, data from a total of 60 healthy young adults (30 females/30 males) were utilized to answer the present research questions (males: age: 26 ± 4 years and body mass index [BMI]: 25 ± 3 kg/m^2^; females: age: 24 ± 3 years and BMI: 24 ± 3 kg/m^2^). From the reliability aspects, data were collected at several time points for each participant. Therefore, all of the unique time points were used as its data point for the concurrent validity aspect. This produced 160 unique data time points across the 60 study participants (52 data sets in females/108 data sets in males) which enabled this investigation to robustly assess the NVC metric concurrent validity objective. Finally, from the accumulation of studies, 11 participants (3 females/8 males) were used for the within-day reliability (age: 26 ± 4 years and BMI: 25 ± 3 kg/m^2^), whereas 17 participants (2 females/15 males) were used for the within-day reliability (age: 26 ± 4 years and BMI: 25 ± 3 kg/m^2^). For the reliability aims, all testing was completed during the early follicular stage (days three to seven) in females experiencing a regular menstrual cycle.^31^ Participants were instructed to abstain from exercise, caffeine, smoking, and alcohol consumption for 12 hours before study commencement. These are widely used guidelines in NVC literature^2^ and/or based upon empirical evidence.^14^ Nutritional intake for the reliability assessments was also controlled, and is published elsewhere.^14^

### Instrumentation

As TCD displays substantial between- and within-sonographer variability,^32^ all assessments occurred in the presence of one of two trained sonographers (JSB and JDS) who had previously administered >1000 individual assessments. The deep conduit vessels of the brain (i.e., middle cerebral artery [MCA] and PCA]) were insonated with TCD via two 2-MHz ultrasound probes (DWL USA, Inc, San Juan Capistrano, CA, USA). The probes were placed over the transtemporal windows and experienced technicians located the right M1 segment of the MCA and the left P1 segment of the PCA. Once located, the vessels were confirmed based upon the expected signal depth and velocity, carotid compressions, and a simple visual task.^1^ The probes were then locked into place using a fitted headpiece, which for the within-day reliability analysis, remained in place across the entirety of the day (DWL USA, Inc, San Juan Capistrano, CA, USA or Spencer Technologies, Seattle, WA, USA). This minimized the impact of sonographer error, as the insonation angle and depth remain unchanged. A 3-lead electrocardiogram was used to capture individual PQRST waveforms, and R-R interval data were calculated using lead II methodology (ADI Instruments, Colorado Springs, CO, USA). A non-invasive, continuous beat-to-beat blood pressure monitoring device was used to capture each vascular pulsatile waveform, which was corrected to the height of the heart using a brachial cuff (Finometer NOVA; Finapres Medical Systems, Amsterdam, The Netherlands or Finometer PRO, Finapres Medical Systems, Amsterdam, Netherlands).^33,34^ Finally, an inline gas analyzer captured breath-to-breath oxygen and carbon dioxide values (ML206; AD Instruments, Colorado Springs, CO, USA). This was calibrated at the start of each data collection with room air (20.93% oxygen, 0.03% carbon dioxide, and 78.08% nitrogen) and a known gas concentration (16% oxygen, 5% carbon dioxide, and balanced nitrogen). All data collected were sampled at 1000 Hz, time-aligned, and stored offline with commercially available software (LabChart Pro Version 8, AD Instruments, Colorado Springs, CO, USA).

### Experimental protocols

Testing took place within either the Cerebrovascular Concussion Laboratory at the University of Calgary or the Sensorimotor Neuroscience and Concussion Laboratory at the University of British Columbia Okanagan. A “*Where’s Waldo?*” complex visual paradigm was used to assess the NVC response, as this has been shown to augment the physiological signal- (i.e., NVC response) -to-physiological-noise (i.e., respiratory sinus arrhythmias, Mayer waves, naturally occurring physiological processes, etc.) ratio and be more engaging.^24,25^ Participants were seated ∼50–60 centimetres away from a 27-inch monitor set to maximum screen brightness. To mitigate the confounding influence of myopia and/or hyperopia, individuals wore corrective eyewear or contacts to ensure all had 20/20 vision. Each participant completed eight trials of ∼20-seconds eyes-closed while sitting quietly followed by ∼40-seconds eyes-open while engaging in the “*Where’s Waldo?*” task.^25^ The eyes-closed duration was adjusted slightly on a trial-by-trial basis to ensure blood pressure and end-tidal values of carbon dioxide (P_ET_CO_2_) were as similar as possible at the start of each task. A new puzzle was presented at the start of each eyes-open trial to promote maximum engagement. For each puzzle, participants were instructed to first find “Waldo”, followed by the secondary characters within the “*Waldo Universe*” (“Wenda”, “Odlaw”, “Wizard Whitebeard”, and “Woof’s Tail”).^35^ No participant was successful at finding all five characters within the 40-seconds.

### Data processing

**Figure 2.**
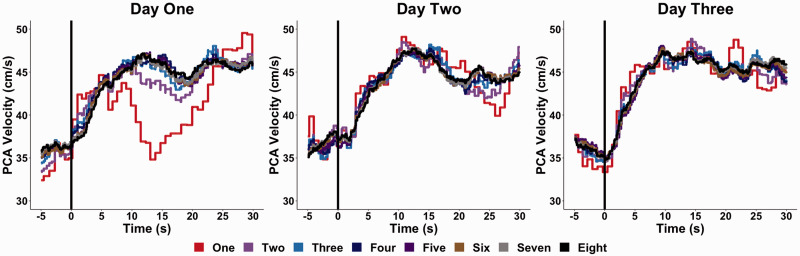
A representative trace of a NVC response derived from one individual across three separate days when averaged across a given number of trials. It is important to note the increasing trials represent the average from the given numbers of trials completed, which would contain the previous trial(s).

Given the temporal resolution of the devices utilized, mean values for systemic arterial pressure, MCA, and PCA were calculated by averaging all data points across each cardiac pulsatile waveform. Further, P_ET_CO_2_ was quantified using the peak partial pressure end-tidal value of carbon dioxide from each breath. Heart rate was calculated through the R-R interval. Artifacts present within the MCA and PCA traces were corrected with a median filter applied within LabChart, which occurred in <0.1% of all trials. All trials were time-aligned to the eyes-open stimulus, producing a total of eight individual trials at each time point for comparison within this investigation. Cleaned data from the trials were extracted in a manner that would resemble data collection occurring on only a given number of trials. Data were extracted on trials ranging from one to eight into self-written Excel scripts (Microsoft, Redmond, Washington, United States). The outcome variables produced were: 1) baseline cerebral blood velocity (CBV) during the 5 seconds prior to the eyes-open stimuli, 2) peak CBV obtained during the first 30 seconds of task engagement following the eyes-open stimulus onset, 3) the relative percent increase between baseline during the eyes closed period and peak CBV during engagement within the “Where’s Waldo?” task, 4) the area-under-the-curve during the first 30 seconds of stimulus onset (AUC30; i.e., total activation), and 5) time-to-peak CBV increase from stimulus onset. The justification for inclusion and a more detailed description of all metrics can be found in Smirl et al.,^25^ and Burma et al.^24^. Although participants engaged in the “*Where Waldo?*” task for 40 seconds, only the first 30 seconds were utilized for a multitude of methodological reasons. Peak CBV will occur ∼20-seconds following the onset of task engagement; however, extending this to 30-seconds ensured that this occurred. More so, the AUC30 relies upon participants completing a minimum of 30-seconds to obtain a valid measure. Having participants engage in the task for 40-seconds eradicates the likelihood of human error by removing the stimulus prematurely, ensuring AUC30 metrics were not artificially blunted. Therefore, the total activation metric is calculated as an area-under-the-curve for all CBV levels relative to the eyes-closed baseline over the first 30-seconds following the eyes-open stimulus (For further details please refer to Figure 2 in literature^14^).

### Sample size calculation

A sample size calculation was conducted *a priori* for both the validity and reliability objectives of the current investigation with G*power (v3.1.9). Using a small, generalized eta squared (n^2^*_G_*) effect size (0.10),^36^ an alpha of 0.05, and a power of 0.80, a sample size of 144 data sets were required to delineate differences between the eight trial groups. However, assessing reliability through group differences has been shown to wash out individual differences, especially when quantifying differences through a binary *p-value.*^37^ Therefore, guidelines set forth by Bonett^38^ were used to determine the sample size for the within- and between-day reliability research questions. For the within-day aim, with an expected reliability of 0.90, precision of 0.10, confidence interval of 95%, and 7 repetitions, a total sample of 9 subjects was required. For the between-day aim, with an expected reliability of 0.90, precision of 0.10, confidence interval of 95%, and 3 repetitions, a total sample of 12 subjects was required. Given these, the present study was adequately powered to answer the *a priori* within- and between-day reliability research aims as datasets from 9 and 17 participants were collected, respectively.

### Statistical analyses

RStudio (version 1.4.1056) was used to analyze the data and compute all inferential and reliability statistics. Previous research has highlighted that biological sex does not confound the NVC metrics of interest;^24^ therefore, data from males and females were amalgamated to increase the power within the present investigation. Regarding the validity objective, Bland-Altman plots with 95% limits of agreement (LOA),^39,40^ adjusted coefficient of determination values (*r*2),^41^ intraclass correlation coefficients (ICC),^42^ and coefficient of variation (CoV)^43^ metrics were computed by comparing trials from one to seven against the “*reference-standard*” eight trials. For all the above metrics, 95% confidence intervals were calculated where inferences were made surrounding the lower and upper limits. The ICC and CoV values were also computed for the within- and between-day reliability aims. As per guidelines by Koo and Li,^42^ ICC and their associated 95% confidence intervals (95% CI) were determined based upon a mean-rating (*k* = 3), absolute-agreement, and 2-way mixed-effects model. Within-participant CoV values were calculated through the quotient of the standard deviation and the mean of all metrics. These values were then averaged across all individuals to determine the CoV mean. Furthermore, the 95% CI for the CoV and *r*^2^ were calculated through a bootstrap approach with 10,000 resamples.^44^ Thresholds for the ICC were based upon the broader literature and set at: <0.50 (poor), 0.50–0.75 (moderate), 0.75–0.90 (good), and >0.90 (excellent).^42^ While there are no agreed-upon thresholds or strict rules to interpret the strength of a *r*^2^ metric,^41^ *a priori* thresholds for the *r*^2^ metrics were determined as: <0.10 (negligible), 0.10–0.30 (small), 0.30–0.50 (moderate), 0.50–0.80 (large), and 0.80–1.00 (very large). Finally, thresholds for the CoV were: <5% (excellent), 5–10% (good), 10–20% (acceptable), and >20% (unacceptable). As the “*Where’s Waldo?*” task has been evidenced to engage the PCA to a greater extent than the MCA,^24,25^ conclusions regarding the optimal number of trials will be centred around the PCA, since it is more relevant for the task utilized. To further understand the concurrent validity, backward stepwise linear regressions were run for each variable to enable the control of various confounding physiological influences (mean arterial pressure, heart rate, respiratory rate, and P_ET_CO_2_). Within each model, trials one-to-seven were compared against eight trials. These confounding influences were removed one at a time, where a change in coefficients between models of greater than 10% was used to delineate an impact of confounding variables.^45,46^ To represent the data, the described validity and reliability metrics are presented comprehensively and transparently for both the PCA and MCA, albeit the latter is detailed within the supplemental material. Data are displayed as mean ± standard deviation or reliability point-estimate ± 95% CI, where appropriate throughout the manuscript. Alpha was set *a priori* at 0.05. For the linear regressions, a significant value was determined when the 95% CI did not contain the null hypothesis of 1.00

## Results

### Physiological and neurovascular coupling outcome metrics

A representative trace for the NVC response within one individual containing one-to-eight trials is displayed in Figure 2. Physiological data (i.e., including heart rate, mean arterial pressure, P_ET_CO_2_, and respiration rate) during the NVC assessment are displayed in Supplemental Table 1 (concurrent validity), Supplemental Table 2 (within-day reliability), and Supplemental Table 3 (between-day reliability). More so, the mean and standard deviation for the NVC outcome variables within both the PCA and MCA for the concurrent validity, within-day reliability, and between-day reliability aims are displayed in Supplemental Table 4, Supplemental Table 5, and Supplemental Table 6, respectively.

### Concurrent validity

**Figure 3.**
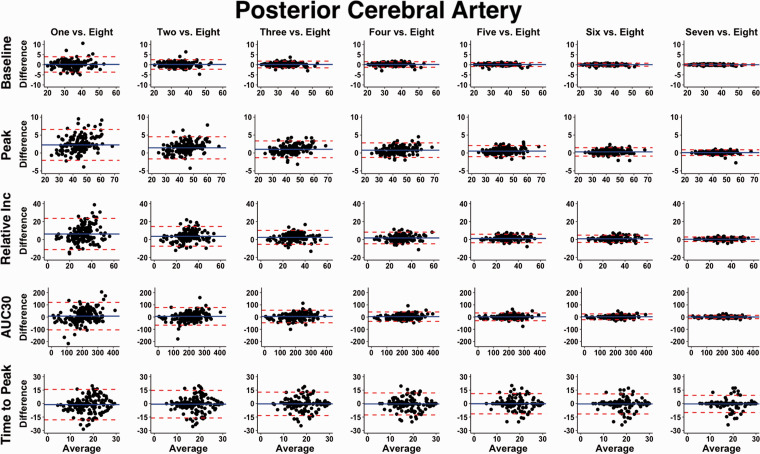
Bland-Altman plots with 95% limits of agreement demonstrating the *concurrent validity* of neurovascular coupling metrics within the posterior cerebral artery (PCA) derived from one to seven trials compared to the “*reference-standard*” eight trials. A total of 160 time points were drawn from 60 participants (30 females/30 males). It is important to note the increasing trials represent the average from the given numbers of trials completed, which would contain the previous trial(s). It should be highlighted the time-to-peak measures displayed wide variance across all trials and therefore this metric should be interpreted with caution. The outcome metrics of interest within the posterior cerebral artery (PCA) included: baseline PCA velocity (cm/s), peak PCA velocity (cm/s), relative percent (%) increase in PCA velocity from baseline to peak, PCA total activation/area-under-the-curve during the first 30-seconds of task engagement (AUC30) (cm/s/30s), and time-to-peak PCA velocity during task engagement (s).

**Figure 4. fig4-0271678X221084400:**
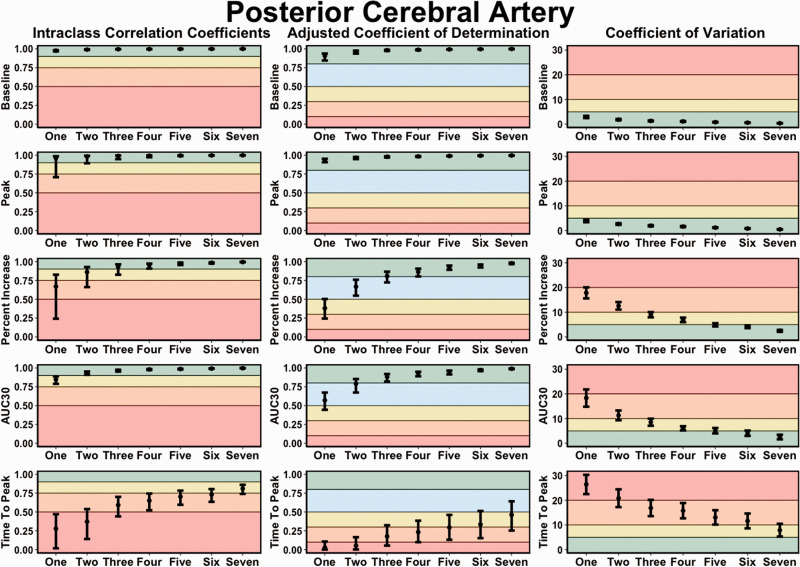
Intraclass correlation coefficients (ICC), adjusted coefficient of determination (*r*^2^) values, and coefficient of variation (COV) metrics demonstrating the *concurrent validity* of neurovascular coupling metrics within the posterior cerebral artery (PCA) derived from one to seven trials compared to the “*reference-standard*” eight trials. It is important to note the increasing trials represent the average from the given numbers of trials completed, which would contain the previous trial(s). Thresholds for the ICC were set at: <0.50 (poor; red), 0.50–0.75 (moderate; orange), 0.75–0.90 (good; yellow), and >0.90 (excellent; green). Thresholds for the adjusted *r*^2^ metrics were set at: <0.10 (negligible; red), 0.10–0.30 (small; orange), 0.30–0.50 (moderate; yellow), 0.50–0.80 (large; blue), and 0.80–1.00 (very large; green). Thresholds for the CoV were: >20% (unacceptable; red), 10–20% (acceptable: orange), 5–10% (good; yellow), and <5% (excellent; green). The outcome metrics of interest within the posterior cerebral artery (PCA) included: baseline PCA velocity (cm/s), peak PCA velocity (cm/s), relative percent (%) increase in PCA velocity from baseline to peak, PCA total activation/area-under-the-curve during the first 30-seconds of task engagement (AUC30) (cm/s/30s), and time-to-peak PCA velocity during task engagement (s).

In general, compared to data obtained from the “*reference-standard*” eight trial condition, the ICC and adjusted *r*^2^ values increased with the completion of more trials, whereas the Bland-Altman 95% LOA and CoV metrics narrowed and decreased with a greater number of trials, respectively (Figure 3). Figure 4 illustrates PCA baseline, peak, percent increase, and AUC_30_ demonstrated excellent ICC estimates (>0.900) when a minimum of four trials were performed. Time-to-peak ICC 95% CI consistently displayed poor, poor-to-moderate, or moderate-to-good across all number of trials (Figure 4). Likewise, four trials were required to achieve very large adjusted *r*^2^ values (>0.800) for the same metrics (i.e., PCA baseline, peak, percent increase, and AUC_30_), whereas time-to-peak predominantly exhibited negligible to moderate *r*^2^ values (Table 1). Finally, Figure 4 shows the CoV continued to decrease with a greater number of trials; however, once four trials were completed, the CoV was <10% for PCA baseline, peak, percent increase, and AUC_30_. Comprehensively, after participants had completed four trials, the NVC values obtained were comparable to values elicited after the completion of eight trials. When physiological covariates were controlled for via backward stepwise linear regressions, three or more trials produced NVC metrics akin to those elicited from eight trials (Table 1). Albeit the estimate and 95% confidence intervals centred around the null of 1.00 to a greater extent with each successive completed trial (Table 1). Respiratory rate, P_ET_CO_2_, heart rate, and mean arterial pressure were all found to confound the NVC response (Table 1). The concurrent validity data for MCA derived NVC metrics are displayed in Supplemental Figure 1 (Bland-Altman plots with 95% limits of agreement) and Supplemental Figure 2 (ICC, adjusted *r*^2^, and CoV metrics).

**Table 1.** Unstandardized regression coefficients with physiological confounders for neurovascular coupling metrics produced during a “*Where’s Waldo?*” paradigm in 60 individuals (30 females/30 males) within the posterior cerebral artery.

	Baseline (cm/s)	Peak (cm/s)	Relative Increase (%)	AUC30 (cm/s/30s)	Time to Peak (s)
One Cycle	1.01 (0.97, 1.05)	1.05 (1.01, 1.10)*	1.21 (1.12, 1.30)*	0.87 (0.74, 1.03)	0.88 (0.81, 0.96)*
Two Cycles	1.01 (0.97, 1.05)	1.03 (0.99, 1.08)	1.12 (1.03, 1.20)*	1.00 (0.85, 1.19)	0.94 (0.86, 1.03)
Three Cycles	1.01 (0.97, 1.05)	1.02 (0.98, 1.07)	1.08 (1.00, 1.17)	1.00 (0.85, 1.18)	0.97 (0.89, 1.06)
Four Cycles	1.01 (0.97, 1.05)	1.02 (0.98, 1.06)	1.06 (0.98, 1.14)	1.00 (0.85, 1.18)	0.97 (0.89, 1.06)
Five Cycles	1.00 (0.97, 1.05)	1.01 (0.97, 1.06)	1.04 (0.96, 1.12)	1.00 (0.85, 1.17)	0.99 (0.91, 1.08)
Six Cycles	1.00 (0.96, 1.04)	1.01 (0.97, 1.05)	1.03 (0.95, 1.11)	1.00 (0.86, 1.16)	0.97 (0.89, 1.06)
Seven Cycles	1.00 (0.96, 1.04)	1.00 (0.96, 1.04)	1.01 (0.94, 1.09)	1.00 (0.87, 1.15)	0.98 (0.90, 1.07)
Confounding Variables	P_ET_CO_2_ and HR	–	MAP and HR	RR, P_ET_CO_2_, and HR	P_ET_CO_2_ and HR

Note: Numerous individuals completed repeated assessments/follow-ups, which produced a total of 160 unique data points. Data were produced from the number of trials completed which ranged from one to eight, which values produced from trials one to seven were compared to the “*reference-standard*” eight trials. Data are beta coefficient (95% confidence interval). Significant values are displayed in bold with an asterisk (*) (*p<0.050*). Area-under-the-curve during the first 30 seconds of stimulus onset (AUC30), centimetre (cm), second (s), percent (%), respiratory rate (RR), partial pressure of carbon dioxide (P_ET_CO_2_), mean arterial pressure (MAP), and heart rate (HR).

### Within-day reliability

**Figure 5. fig5-0271678X221084400:**
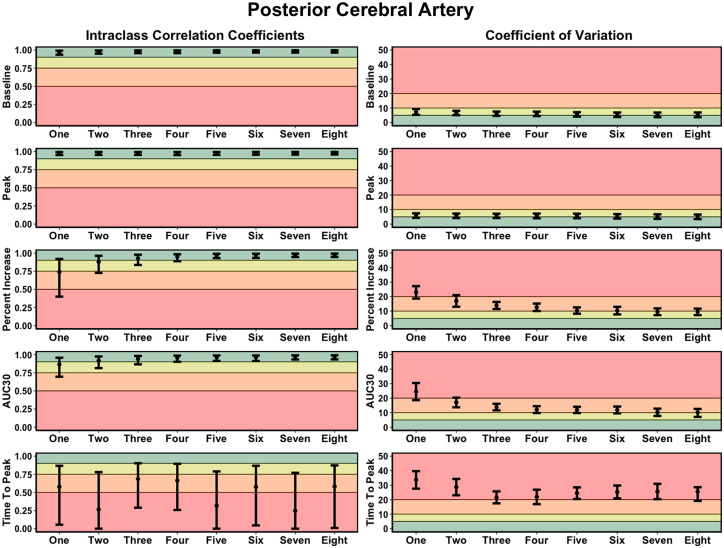
Intraclass correlation coefficients (ICC) and coefficient of variation (COV) metrics demonstrating the *within-day reliability* of neurovascular coupling metrics within the posterior cerebral artery (PCA) derived from one to eight trials. It is important to note the increasing trials represent the average from the given numbers of trials completed, which would contain the previous trial(s). Thresholds for the ICC were set at: <0.50 (poor; red), 0.50–0.75 (moderate; orange), 0.75–0.90 (good; yellow), and >0.90 (excellent; green). Thresholds for the CoV were: >20% (unacceptable; red), 10–20% (acceptable: orange), 5–10% (good; yellow), and <5% (excellent; green). The outcome metrics of interest within the posterior cerebral artery (PCA) included: baseline PCA velocity (cm/s), peak PCA velocity (cm/s), relative percent (%) increase in PCA velocity from baseline to peak, PCA total activation/area-under-the-curve during the first 30-seconds of task engagement (AUC30) (cm/s/30s), and time-to-peak PCA velocity during task engagement (s).

Good-to-excellent ICC values were produced with three or more completed NVC trials for the main four variables previously discussed; however, with five or more trials, the lower and upper 95% CI were classified as excellent (Figure 5). Three trials were required to produce acceptable-to-good levels of variation, which continually decreased with a greater number of trials completed (Figure 5). Finally, the time-to-peak metrics generally displayed poor-to-moderate ICC values with unacceptable levels of CoV (Figure 5). Conclusively, the NVC response demonstrated excellent within-day reliability when five or more trials were completed (Figure 5). The within-day reliability metrics for all MCA metrics produced during the “Where’s Waldo?” paradigm are displayed in Supplemental Figure 3 (ICC and CoV metrics).

### Between-day reliability

**Figure 6.**
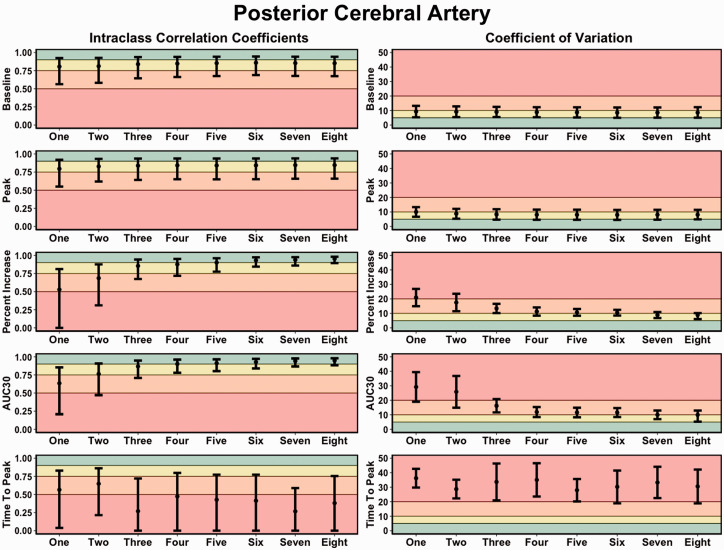
Intraclass correlation coefficients (ICC) and coefficient of variation (COV) metrics demonstrating the *between-day reliability* of neurovascular coupling metrics within the posterior cerebral artery (PCA) derived from one to eight trials. It is important to note the increasing trials represent the average from the given numbers of trials completed, which would contain the previous trial(s). Thresholds for the ICC were set at: <0.50 (poor; red), 0.50–0.75 (moderate; orange), 0.75–0.90 (good; yellow), and >0.90 (excellent; green). Thresholds for the CoV were: >20% (unacceptable; red), 10–20% (acceptable: orange), 5–10% (good; yellow), and <5% (excellent; green). The outcome metrics of interest within the posterior cerebral artery (PCA) included: baseline PCA velocity (cm/s), peak PCA velocity (cm/s), relative percent (%) increase in PCA velocity from baseline to peak, PCA total activation/area-under-the-curve during the first 30-seconds of task engagement (AUC30) (cm/s/30s), and time-to-peak PCA velocity during task engagement (s).

For baseline and peak PCA metrics, all trials displayed moderate-to-excellent levels of reliability, whereas, for percent increase and AUC_30_, five trials elicited good-to-excellent levels of reliability (Figure 6). Further, the CoV generally rated from acceptable-to-good after the completion of four trials (Figure 6). While the between-day reliability demonstrated slightly greater variability, reliable estimates were able to be produced with the completion of a minimum of five trials (Figure 6). The between-day reliability NVC metrics derived within the MCA are displayed in Supplemental Figure 4 (ICC and CoV metrics).

## Discussion

The current standardized practice regarding NVC literature has suggested studies include five-to-ten trials of a given task to ensure an adequate signal-to-noise ratio is captured.^2^ However, this recommendation has not been rigorously assessed based upon the ability to derive physiological valid and reliable estimates. Therefore, the present study sought to address this gap within the literature by comparing the concurrent validity (Figures 3 and 4), within-day reliability (Figure 5), and between-day reliability (Figure 6) from trials ranging from one-to-eight during a complex visual task (i.e., “*Where’s Waldo?*”). In general, the validity and reliability of the NVC metrics increased with each successive trial completed. However, based upon the statistical guidelines widely used across the broader scientific literature,^38–44^ the overall findings from this investigation reveal a minimum of five trials is needed to produce reliable estimates. There nonetheless are three important caveats that must be considered in light of these results. First, these findings were based upon a task designed to maximize the NVC response, which may not be transferable to other methodological approaches that do not result in the same neurovascular activation (e.g., reading, viewing simple shapes, etc.). Second, while five trials produced reliable and valid results, studies seeking to discern pathophysiological changes within clinical populations may require greater than this. Until this second consideration is delineated, the use of five trials should be used as a salvaging technique and not as an *a priori* methodological plan. Third, the between-day reliability was lower compared to the within-day reliability due to methodological differences (e.g., removal of TCD headframe between days, slightly different insonation angle or depth, etc.).

### Physiological underpinnings

The current investigation demonstrated NVC metrics increased in validity (Figures 3 and 4) and reliability (Figures 5 and 6) when a greater number of trials were utilized. The explanation for this likely underlies the fact the NVC response is dependent upon a complex process of neuronal signalling.^2^ This increases the likelihood the NVC response may be confounded by systemic blood pressure or other physiological processes (e.g., Mayer waves, respiratory sinus arrhythmia, etc.), which was demonstrated by the linear regression analyses (Table 1). With the completion of each successive trial, the impact of physiologically confounding variables on the outcome measure of interest is further mitigated as the signal-to-noise ratio is enhanced (Figures 3 and 4). Therefore, while the findings regarding the number of trials required for valid results may be dependent upon the methodological utilized (“*Where’s Waldo?*”), it nonetheless is universal that studies need to measure and control for these highlighted physiological confounders (i.e., blood pressure, heart rate, carbon dioxide levels) to avoid biased NVC estimates.

It should also be noted within this investigation, the trials that had clear systemic blood pressure influence (Mayer waves)^47^ were included. The reliability between days and time points may be further augmented if each trial is assessed on a trial-by-trial basis, where trials predominantly mediated by systemic blood pressure and/or arterial carbon dioxide are excluded. Hence, with the completion of eight trials and an *a priori* criteria of trials to exclude based upon physiological confounder influences, researchers would still have sufficient data to achieve a high signal-to-noise ratio regarding the NVC response.

Furthermore, NVC has demonstrated clinical utility being able to delineate differences within numerous clinical conditions including: Fabry Disease,^48^ sport-related concussion,^7^ hyperhomocysteinemia,^49^ pulmonary hypertension,^5^ ischemic stroke,^4^ multiple sclerosis,^6^ spinal cord injury,^50^ among others. While the present findings displayed a minimum of five trials produce reproducible estimates (Figures 3 [Fig fig4-0271678X221084400][Fig fig5-0271678X221084400]to 6), to maximally understand the pathogenesis and adequately diagnose these conditions the most robust methodological practices should be utilized. This ideally should include eight trials of a maximally engaging task.^2,24,25^ However, some clinical populations may experience fatigue and/or a worsening of symptomology during tasks that are easily completed within healthy and/or control populations. As it is common practice in cerebrovascular research to try to obtain as much physiological relevant data as possible (e.g., NVC, dynamic cerebral autoregulation, cerebrovascular reactivity, etc.), a trade-off exists with being able to obtain meaningful and comprehensive data while not overburdening participants. For example, within an acutely or chronically concussed population, individuals may experience light sensitivity and could potentially become symptomatic with the completion of eight-to-ten trials. Conclusively, while the best methodological practice is to collect a total of eight NVC trials, researchers should consider the other tasks clinical populations may complete over the course of the testing battery and incorporate more rest time between tasks and/or only require participants to complete five NVC trials.

### Methodological considerations for future investigations

As previously stated, the reliability was lower for the between-day analysis (Figure 6) compared to the within-day analysis (Figure 5). This likely stems from both sonographer and naturally occurring physiological differences, which are factors researchers should be cognizant of when developing their methodological approaches. The within-day protocol consisted of seven time points, where the headframe remained secure and the angle and depth of insonation did not change. Conversely, the between-day protocol examined three separate time points, where the headframe was removed, and the probes reapplied. The latter analysis could have been impacted by a slight temporal difference in probe placement and/or angle of insonation, where even a minuscule difference could impact the reliability. More so, inter-rater reliability has been demonstrated to be impacted by the experience of a sonographer, as well as when using multiple sonographers within a single study.^32^ In the current study, one of two highly trained sonographers were present at every data collection to minimize the likelihood this influenced the present results. Finally, compared to the within-day assessments, the between-day analysis would more likely be impacted by various factors, including but not limited to stress levels, weather pattern changes, sleep quality, and so forth. Strategies that can help mitigate these influences for between-day assessments include: 1) minimizing the number of sonographers insonating vessels in a given study, 2) upon finding an artery, recording the depth of insonation and baseline velocity, 3) seeking to insonate an artery with a bifurcation from other arteries, and 4) having participants complete validated questionnaires to see if deviations can be attributed to various physiological fluctuations. Therefore, as a greater likelihood exists of between-day assessments experiencing confounding influences, studies looking to conduct longitudinal assessments may benefit from completing more than five trials.

It is imperative to note the three investigations used in the present study only reported the individual averaged NVC data across each of the eight eyes-open, eyes-closed trials of data collection. Therefore, the current study will be insufficient to ascertain the complete validity and reliability associated across the entire recommendation window of five-to-ten eyes-open, eyes-closed trials, as previously proposed.^2^ More specifically, this investigation is unable to determine if performing nine or ten trials will yield more valid and reliable results than five-to-eight trials. Nonetheless, the current study includes a robust data set and is extremely well-powered to investigate whether eight trials are adequate in guiding future studies. These findings should be taken in light of the specific methodological approach used (i.e., “*Where’s Waldo?*” search). A “*Golden Rule*” of the required number of trials may be unrealistic due to the divergence within the literature surrounding quantifying NVC using various tasks (e.g., reading, finger tapping, etc.) and with other neuroimaging equipment (e.g., functional near-infrared spectroscopy, functional magnetic resonance imaging, etc.). Nonetheless, researchers can use this as a template to scrutinize their methodological approaches to ensure they are scientifically valid and reliable.

### Limitations

A limitation of the current investigation is the use of TCD independently to assess the NVC response, which has also been quantified using other imaging techniques such as functional near-infrared spectroscopy, functional magnetic resonance imaging, and so forth. Nevertheless, a benefit of TCD is the temporal resolution (i.e., ∼200 Hz), where the functionality of the cerebrovasculature can be quantified with austerity.^51^ However, TCD is not without limitations. Because the diameter of the insonated vessel cannot be quantified, a supposition that diameter does not change during a task must be made.^9^ Previous research with high functional magnetic resonance imaging compared diameter changes when participants remained within eucapnic levels (∼35 to ∼45 mmHg).^9^ When eucapnic, valid estimates can be drawn with TCD^9^, which was the case within the current investigation. More so, Table 1 demonstrates that when physiological covariates are controlled, NVC estimates increase in scientific validity. It is imperative to note that the task utilized was a complex visual paradigm that evoked a greater response within the PCA compared to the MCA. The number of trials may slightly deviate if cognitive (e.g., n-back, memory tasks, etc.^52–57^) or motor (e.g., finger tapping, arm raises, etc.^58,59^) tasks are used, as they may not produce a similar response within the MCA. Nevertheless, the present results highlight a foundation future research investigations can build upon to ensure robust and reliable methodological approaches are utilized across the field. Lastly, it is important to consider an individual’s tiredness could impact NVC metrics. For example, if an individual becomes tired and loses interest in a task, they may have a blunted NVC response. To counteract this, it is recommended researchers use a maximally engaging task and/or complete eight trials over the course of two blocks with rest in between.^24^

### Conclusions

In summary, this investigation sought to delineate how NVC metrics are impacted by the number of trials completed, as previous recommendations from Phillips et al.,^2^ stated five-to-ten trials should be performed. The current findings demonstrated future studies utilizing TCD to quantify the NVC response within the PCA should ideally have participants complete no less than five trials when using a “*Where’s Waldo?*” paradigm. However, the precise number depends upon the research question at hand, where a “*Golden Rule*” may not be entirely transferrable. As an illustration, five trials may be sufficient for a study seeking to answer a methodological question in healthy individuals. However, five may not be adequate for longitudinal studies and/or clinical investigations that are seeking to delineate pathophysiological differences of various conditions and diseases. Therefore, it is recommended that the use of five trials should be employed only as a salvaging technique or within populations where successive repetitions could induce clinical symptoms. These results are imperative for studies seeking to delineate subtle differences between healthy and clinical populations, as using improper methodology may conceal physiological discrepancies between groups. Conclusively, as a “*one-size-fits-all*” recommendation may be inappropriate, researchers should scrutinize their methodological techniques to ensure they are using scientifically sound approaches.

## Supplemental Material

sj-jpg-1-jcb-10.1177_0271678X221084400 - Supplemental material for Neurovascular coupling on trial: How the number of trials completed impacts the accuracy and precision of temporally derived neurovascular coupling estimates Click here for additional data file. Supplemental material, sj-jpg-1-jcb-10.1177_0271678X221084400 for Neurovascular coupling on trial: How the number of trials completed impacts the accuracy and precision of temporally derived neurovascular coupling estimates by Joel S Burma, Rowan K Van Roessel, Ibukunoluwa K Oni, Jeff F Dunn and Jonathan D Smirl in Journal of Cerebral Blood Flow & Metabolism

sj-jpg-2-jcb-10.1177_0271678X221084400 - Supplemental material for Neurovascular coupling on trial: How the number of trials completed impacts the accuracy and precision of temporally derived neurovascular coupling estimates Click here for additional data file. Supplemental material, sj-jpg-2-jcb-10.1177_0271678X221084400 for Neurovascular coupling on trial: How the number of trials completed impacts the accuracy and precision of temporally derived neurovascular coupling estimates by Joel S Burma, Rowan K Van Roessel, Ibukunoluwa K Oni, Jeff F Dunn and Jonathan D Smirl in Journal of Cerebral Blood Flow & Metabolism

sj-jpg-3-jcb-10.1177_0271678X221084400 - Supplemental material for Neurovascular coupling on trial: How the number of trials completed impacts the accuracy and precision of temporally derived neurovascular coupling estimates Click here for additional data file. Supplemental material, sj-jpg-3-jcb-10.1177_0271678X221084400 for Neurovascular coupling on trial: How the number of trials completed impacts the accuracy and precision of temporally derived neurovascular coupling estimates by Joel S Burma, Rowan K Van Roessel, Ibukunoluwa K Oni, Jeff F Dunn and Jonathan D Smirl in Journal of Cerebral Blood Flow & Metabolism

sj-pdf-4-jcb-10.1177_0271678X221084400 - Supplemental material for Neurovascular coupling on trial: How the number of trials completed impacts the accuracy and precision of temporally derived neurovascular coupling estimates Click here for additional data file. Supplemental material, sj-pdf-4-jcb-10.1177_0271678X221084400 for Neurovascular coupling on trial: How the number of trials completed impacts the accuracy and precision of temporally derived neurovascular coupling estimates by Joel S Burma, Rowan K Van Roessel, Ibukunoluwa K Oni, Jeff F Dunn and Jonathan D Smirl in Journal of Cerebral Blood Flow & Metabolism

sj-pdf-5-jcb-10.1177_0271678X221084400 - Supplemental material for Neurovascular coupling on trial: How the number of trials completed impacts the accuracy and precision of temporally derived neurovascular coupling estimates Click here for additional data file. Supplemental material, sj-pdf-5-jcb-10.1177_0271678X221084400 for Neurovascular coupling on trial: How the number of trials completed impacts the accuracy and precision of temporally derived neurovascular coupling estimates by Joel S Burma, Rowan K Van Roessel, Ibukunoluwa K Oni, Jeff F Dunn and Jonathan D Smirl in Journal of Cerebral Blood Flow & Metabolism

sj-jpg-6-jcb-10.1177_0271678X221084400 - Supplemental material for Neurovascular coupling on trial: How the number of trials completed impacts the accuracy and precision of temporally derived neurovascular coupling estimates Click here for additional data file. Supplemental material, sj-jpg-6-jcb-10.1177_0271678X221084400 for Neurovascular coupling on trial: How the number of trials completed impacts the accuracy and precision of temporally derived neurovascular coupling estimates by Joel S Burma, Rowan K Van Roessel, Ibukunoluwa K Oni, Jeff F Dunn and Jonathan D Smirl in Journal of Cerebral Blood Flow & Metabolism
