# Supplementary figures and images for: Neurovascular coupling on trial: How the number of trials completed impacts the accuracy and precision of temporally derived neurovascular coupling estimates
Source: J Cereb Blood Flow Metab. 2022 Feb 25;42(8):1478–92. doi: 10.1177/0271678X221084400 (PMC9274868; doi:10.1177/0271678X221084400)

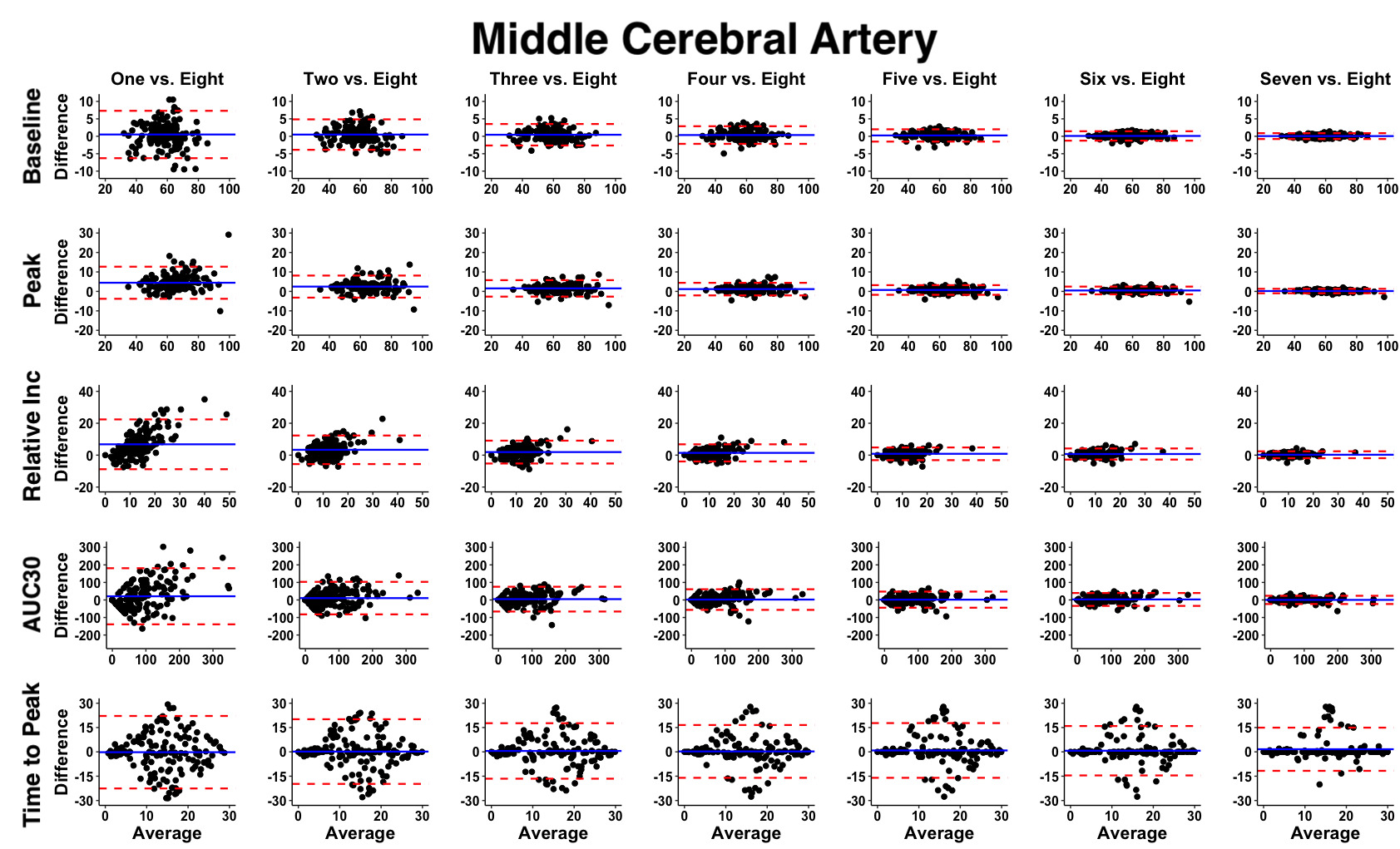

Supplement: sj-jpg-1-jcb-10.1177_0271678X221084400 - Supplemental material for Neurovascular coupling on trial: How the number of trials completed impacts the accuracy and precision of temporally derived neurovascular coupling estimates [file sj-jpg-1-jcb-10.1177_0271678X221084400.jpg]

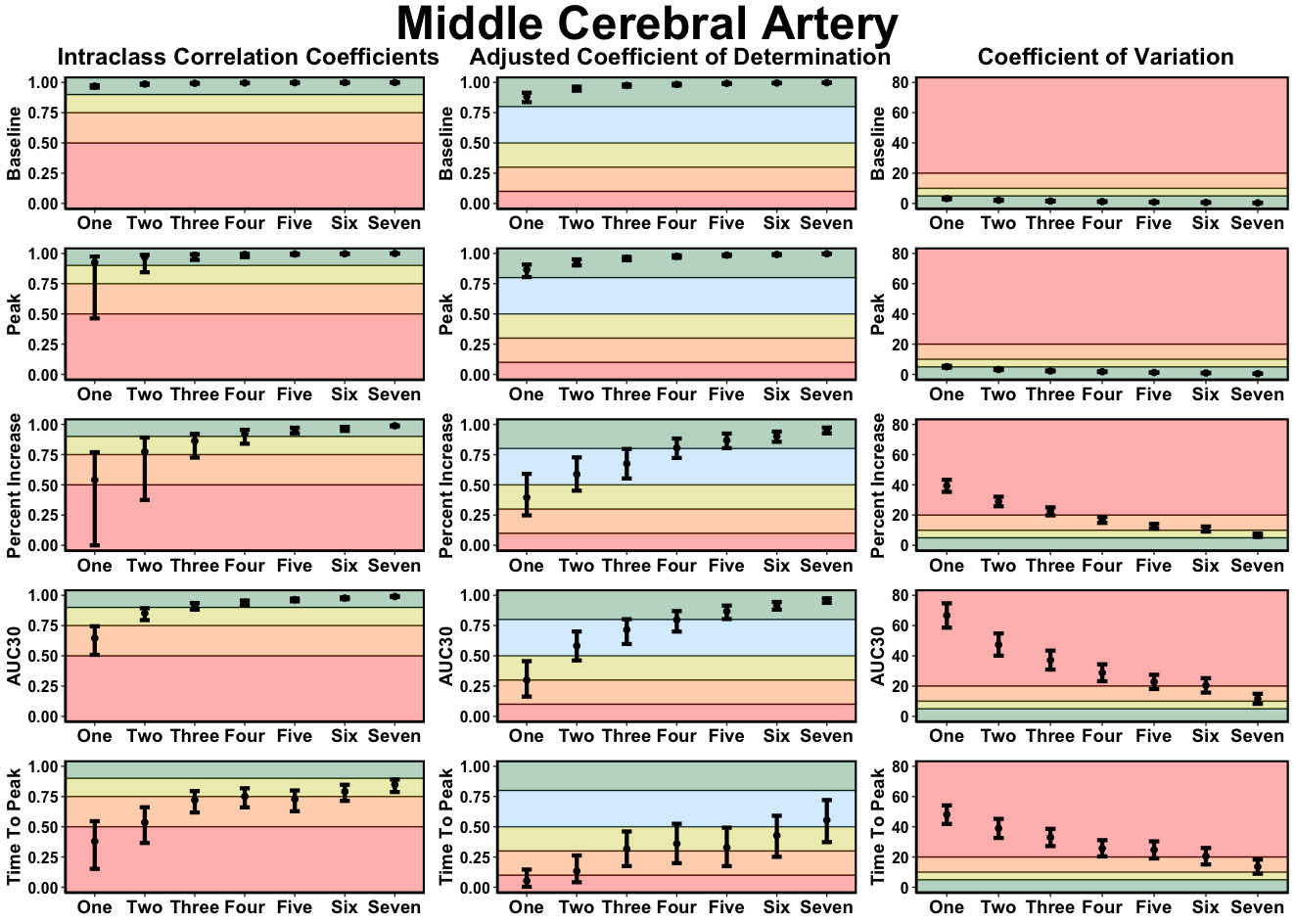

Supplement: sj-jpg-2-jcb-10.1177_0271678X221084400 - Supplemental material for Neurovascular coupling on trial: How the number of trials completed impacts the accuracy and precision of temporally derived neurovascular coupling estimates [file sj-jpg-2-jcb-10.1177_0271678X221084400.jpg]

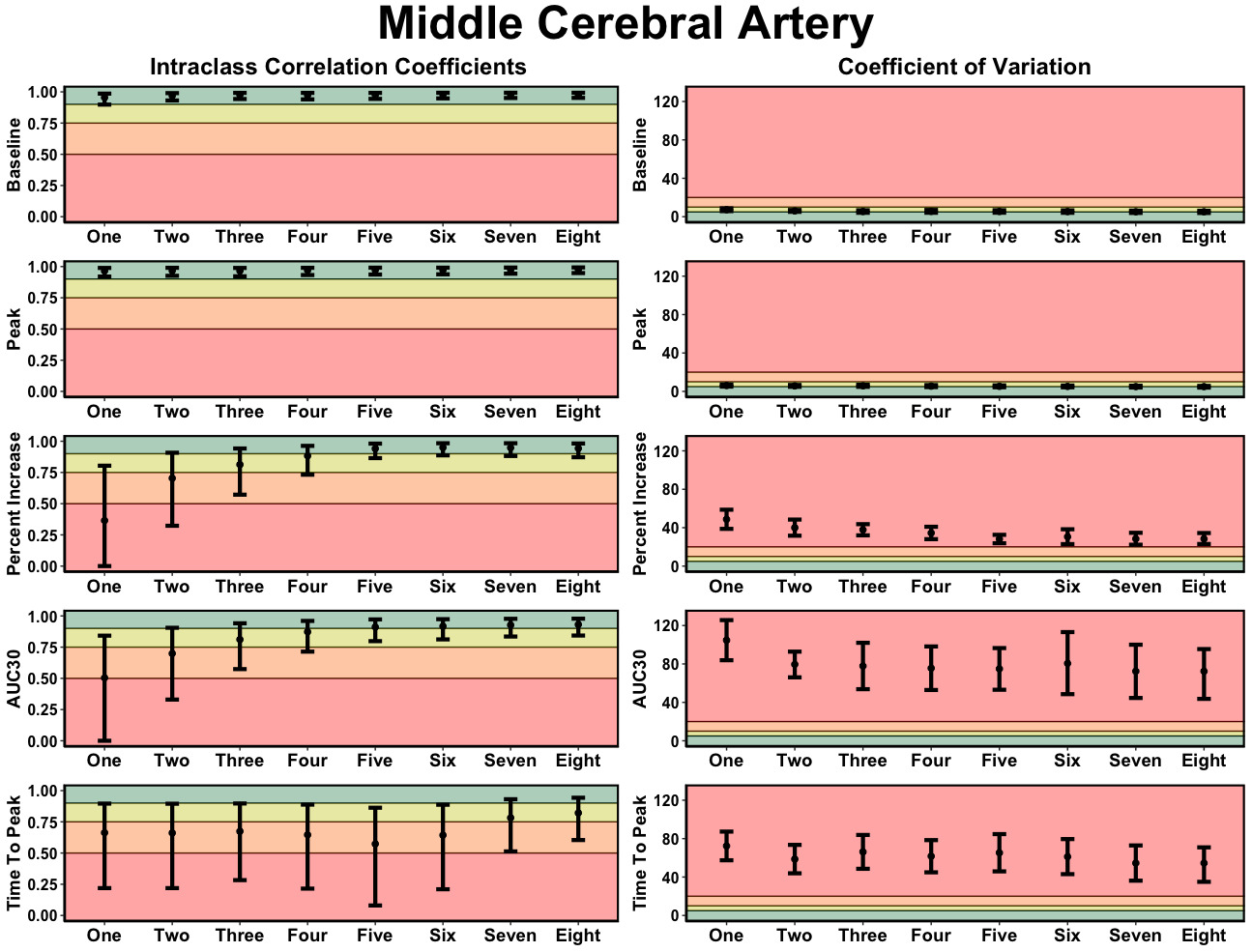

Supplement: sj-jpg-3-jcb-10.1177_0271678X221084400 - Supplemental material for Neurovascular coupling on trial: How the number of trials completed impacts the accuracy and precision of temporally derived neurovascular coupling estimates [file sj-jpg-3-jcb-10.1177_0271678X221084400.jpg]

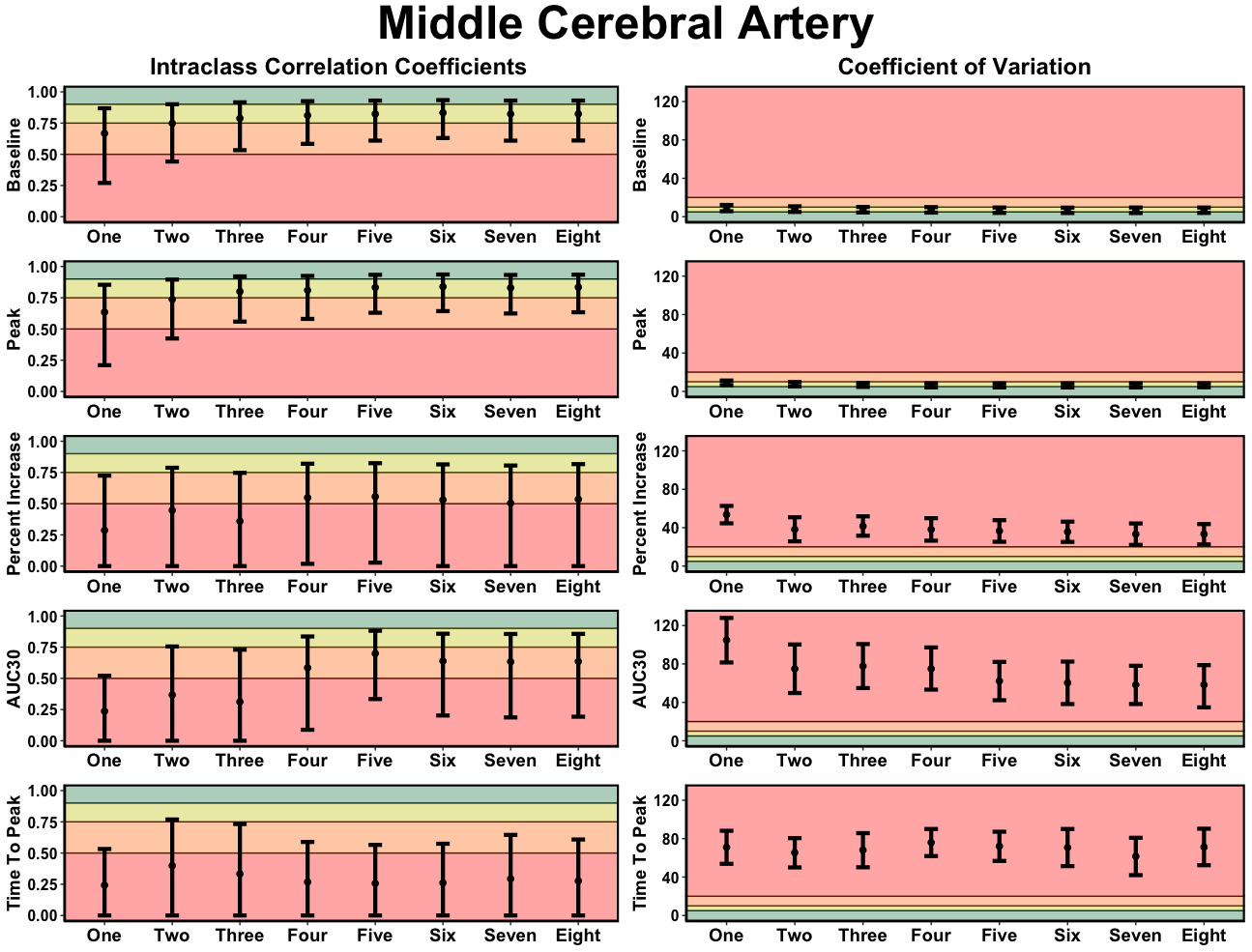

Supplement: sj-jpg-6-jcb-10.1177_0271678X221084400 - Supplemental material for Neurovascular coupling on trial: How the number of trials completed impacts the accuracy and precision of temporally derived neurovascular coupling estimates [file sj-jpg-6-jcb-10.1177_0271678X221084400.jpg]
