# Supplementary material for: Neurovascular coupling on trial: How the number of trials completed impacts the accuracy and precision of temporally derived neurovascular coupling estimates
Source: J Cereb Blood Flow Metab. 2022 Feb 25;42(8):1478–92. doi: 10.1177/0271678X221084400 (PMC9274868; doi:10.1177/0271678X221084400)
Supplement: sj-pdf-4-jcb-10.1177_0271678X221084400 - Supplemental material for Neurovascular coupling on trial: How the number of trials completed impacts the accuracy and precision of temporally derived neurovascular coupling estimates [file sj-pdf-4-jcb-10.1177_0271678X221084400.pdf]

Supplemental Table 1: Physiological data during a "*Where's Waldo?*" paradigm in 60 individuals (30 females / 30 males). Numerous individuals completed repeated assessments/follow-ups, which produced a total of 160 unique data points. Data were produced from the number of trials completed which ranged from one to eight.

**Variable**	**One**	**Two**	**Three**	**Four**	**Five**	**Six**	**Seven**	**Eight**
$P_{ET}CO_2$	38.0 ± 2.7	38.2 ± 2.6	38.2 ± 2.6	38.2 ± 2.6	38.2 ± 2.6	38.2 ± 2.6	38.2 ± 2.6	38.2 ± 2.6
Respiratory Rate	14.7 ± 3.3	14.7 ± 3.0	14.7 ± 2.8	14.7 ± 2.8	14.7 ± 2.7	14.7 ± 2.7	14.7 ± 2.6	14.7 ± 2.6
Mean Arterial Pressure	90.9 ± 10.2	90.4 ± 10.2	90.3 ± 10.1	90.2 ± 10.0	90.1 ± 10.0	90.1 ± 10.0	90.1 ± 9.9	90.2 ± 9.9
Heart Rate	67.2 ± 10.7	67.8 ± 10.6	68.1 ± 10.5	68.3 ± 10.5	68.5 ± 10.5	68.7 ± 10.5	68.8 ± 10.5	69.0 ± 10.5

Data are displayed as mean ± standard deviation. Partial pressure end-tidal values of carbon dioxide ($P_{ET}CO_2$).

Supplemental Table 2: Physiological data during a "*Where's Waldo?*" paradigm in 11 individuals (3 females / 8 males) at seven time points across a single day. Data were produced from the number of trials completed which ranged from one to eight.

Variable	Time Point	One	Two	Three	Four	Five	Six	Seven	Eight
$P_{ET}CO_2$	08:00	39.1 ± 1.8	39.3 ± 1.9	39.4 ± 1.7	39.4 ± 1.7	39.4 ± 1.6	39.3 ± 1.5	39.4 ± 1.5	39.3 ± 1.5
$P_{ET}CO_2$	09:30	38.6 ± 1.7	38.8 ± 1.3	38.7 ± 1.5	38.7 ± 1.4	38.5 ± 1.5	38.5 ± 1.5	38.4 ± 1.6	38.4 ± 1.5
$P_{ET}CO_2$	10:30	37.8 ± 2.7	37.8 ± 2.5	37.9 ± 2.5	37.9 ± 2.2	38.0 ± 2.2	37.9 ± 2.3	37.9 ± 2.3	37.9 ± 2.3
$P_{ET}CO_2$	11:30	36.8 ± 2.5	37.1 ± 2.5	37.2 ± 2.4	37.2 ± 2.4	37.2 ± 2.6	37.1 ± 2.6	37.1 ± 2.6	37.1 ± 2.6
$P_{ET}CO_2$	13:30	39.5 ± 2.0	39.7 ± 2.1	39.8 ± 2.1	39.8 ± 2.0	39.8 ± 1.9	39.7 ± 1.9	39.7 ± 1.8	39.6 ± 1.8
$P_{ET}CO_2$	15:30	39.0 ± 2.3	39.1 ± 2.6	39.1 ± 2.4	39.1 ± 2.4	39.1 ± 2.3	39.1 ± 2.4	39.0 ± 2.4	39.1 ± 2.4
$P_{ET}CO_2$	17:30	38.2 ± 2.3	38.3 ± 2.2	38.4 ± 2.0	38.4 ± 2.0	38.4 ± 1.9	38.3 ± 1.9	38.3 ± 2.0	38.2 ± 2.0
Respiratory Rate	08:00	15.1 ± 3.1	13.8 ± 1.7	13.7 ± 1.5	13.7 ± 1.5	13.7 ± 1.4	13.6 ± 1.5	13.6 ± 1.5	13.6 ± 1.4
Respiratory Rate	09:30	14.1 ± 2.8	14.1 ± 2.4	14.4 ± 2.1	14.4 ± 2.2	14.5 ± 2	14.5 ± 1.9	14.5 ± 1.8	14.4 ± 1.8
Respiratory Rate	10:30	14.4 ± 2.4	14.8 ± 2.4	14.8 ± 1.8	14.8 ± 1.7	15.0 ± 1.7	15.0 ± 1.6	15.0 ± 1.6	14.9 ± 1.5
Respiratory Rate	11:30	14.6 ± 2.4	15.0 ± 2.3	14.9 ± 2.0	14.8 ± 2.0	14.7 ± 2.0	14.8 ± 2.0	14.7 ± 2.0	14.8 ± 2.1
Respiratory Rate	13:30	15.0 ± 2.8	15.0 ± 2.4	15.2 ± 2.2	15.4 ± 2.0	15.4 ± 1.9	15.6 ± 1.8	15.6 ± 1.7	15.5 ± 1.7
Respiratory Rate	15:30	15.4 ± 2.8	15.5 ± 2.7	15.4 ± 2.7	15.5 ± 2.7	15.4 ± 2.5	15.4 ± 2.5	15.5 ± 2.5	15.4 ± 2.4
Respiratory Rate	17:30	15.3 ± 2.5	15.3 ± 2.4	15.1 ± 2.1	15.1 ± 2.1	15.1 ± 2.1	15.1 ± 2.0	15.1 ± 2.0	15.2 ± 1.9
Mean Arterial Pressure	08:00	90.1 ± 11.6	88.5 ± 11.6	87.3 ± 11.1	87.3 ± 10.9	87.0 ± 10.8	86.9 ± 10.5	86.9 ± 10.4	86.8 ± 10.3
Mean Arterial Pressure	09:30	91.2 ± 9.3	90.9 ± 8.7	90.8 ± 8.3	90.6 ± 8.3	90.2 ± 8.5	90.1 ± 8.6	90.1 ± 8.7	90.2 ± 8.8
Mean Arterial Pressure	10:30	97.0 ± 8.7	96.8 ± 9.1	96.9 ± 8.7	96.9 ± 9.0	97.0 ± 9.1	96.8 ± 9.1	96.7 ± 9.2	96.6 ± 9.3
Mean Arterial Pressure	11:30	94.9 ± 6.1	96.0 ± 6.4	96.4 ± 6.8	96.2 ± 7.0	95.8 ± 7.6	96.0 ± 7.9	96.0 ± 8.1	96.2 ± 8.4
Mean Arterial Pressure	13:30	92.5 ± 6.4	91.7 ± 8.7	91.8 ± 8.3	91.6 ± 8.2	91.7 ± 7.9	91.5 ± 7.7	91.5 ± 7.7	91.6 ± 7.7
Mean Arterial Pressure	15:30	93.2 ± 5.0	92.9 ± 5.2	92.7 ± 5.7	92.7 ± 5.6	92.7 ± 6.0	93.0 ± 5.9	93.0 ± 5.9	93.0 ± 5.9
Mean Arterial Pressure	17:30	94.2 ± 8.5	94.3 ± 8.0	94.3 ± 8.3	94.6 ± 8.3	94.9 ± 8.5	95.1 ± 8.5	95.3 ± 8.5	95.5 ± 8.4
Heart Rate	08:00	68.4 ± 7.4	68.6 ± 7.3	69.0 ± 7.4	69.1 ± 7.5	69.3 ± 7.8	69.2 ± 7.7	69.2 ± 7.8	69.2 ± 7.7
Heart Rate	09:30	61.3 ± 7.9	62.7 ± 8.2	63.2 ± 8.2	63.5 ± 8.3	63.9 ± 8.4	64.2 ± 8.3	64.4 ± 8.2	64.6 ± 8.2
Heart Rate	10:30	62.5 ± 10.9	63.4 ± 10.8	63.8 ± 10.6	63.9 ± 10.7	64 ± 10.8	64.3 ± 11.0	64.4 ± 11.0	64.7 ± 11.0
Heart Rate	11:30	63.1 ± 12.2	64.2 ± 11.8	64.5 ± 11.4	64.6 ± 11.3	64.9 ± 11.2	64.9 ± 11.2	65.2 ± 11.2	65.3 ± 11.2
Heart Rate	13:30	72.3 ± 8.1	73.0 ± 8.4	73.4 ± 8.6	73.5 ± 8.5	73.5 ± 8.6	73.6 ± 8.7	73.7 ± 8.7	73.9 ± 8.8
Heart Rate	15:30	66.6 ± 11.0	67.3 ± 11	67.4 ± 10.8	67.8 ± 10.9	67.8 ± 10.9	68.0 ± 10.8	68.1 ± 10.6	68.1 ± 10.6
Heart Rate	17:30	64.2 ± 10.3	64.5 ± 10.4	64.7 ± 10.4	65.0 ± 10.6	65.1 ± 10.5	65.3 ± 10.1	65.6 ± 9.9	65.7 ± 10.0

Data are displayed as mean ± standard deviation. Partial pressure end-tidal values of carbon dioxide ($P_{ET}CO_2$).

Supplemental Table 3: Physiological data during a "*Where's Waldo?*" paradigm in 17 individuals (3 females / 14 males) at three time points on separate days. Data were produced from the number of trials completed which ranged from one to eight.

Variable	Time Point	One	Two	Three	Four	Five	Six	Seven	Eight
$P_{ET}CO_2$	Day One	38.5 ± 2.6	38.6 ± 2.9	38.7 ± 2.9	38.6 ± 3.2	38.6 ± 3.2	38.5 ± 3.2	38.5 ± 3.3	38.5 ± 3.3
$P_{ET}CO_2$	Day Two	38.7 ± 2.4	38.7 ± 2.5	38.5 ± 2.6	38.5 ± 2.8	38.5 ± 2.7	38.5 ± 2.8	38.5 ± 2.8	38.5 ± 2.8
$P_{ET}CO_2$	Day Three	38.5 ± 2.5	38.4 ± 2.3	38.4 ± 2.4	38.4 ± 2.5	38.3 ± 2.4	38.3 ± 2.4	38.2 ± 2.4	38.2 ± 2.3
Respiratory Rate	Day One	14.6 ± 2.2	14.3 ± 1.9	14.4 ± 1.9	14.3 ± 1.8	14.4 ± 1.8	14.4 ± 1.9	14.3 ± 1.8	14.3 ± 1.8
Respiratory Rate	Day Two	15.4 ± 3.5	15.1 ± 3.3	15.1 ± 3.4	14.8 ± 3.4	14.8 ± 3.4	14.7 ± 3.3	14.7 ± 3.3	14.7 ± 3.3
Respiratory Rate	Day Three	15.2 ± 2.9	15.2 ± 2.3	15.2 ± 2.1	15.2 ± 2.0	15.3 ± 2.0	15.2 ± 1.9	15.3 ± 1.9	15.2 ± 1.9
Mean Arterial Pressure	Day One	89.6 ± 10.2	88.7 ± 9.9	88.1 ± 9.8	88.1 ± 9.5	87.9 ± 9.5	87.9 ± 9.3	87.9 ± 9.3	87.8 ± 9.2
Mean Arterial Pressure	Day Two	88.2 ± 8.1	87.7 ± 8.0	87.8 ± 8.3	87.6 ± 8.0	87.6 ± 7.6	87.7 ± 7.6	87.8 ± 7.3	88.1 ± 7.3
Mean Arterial Pressure	Day Three	91.9 ± 8.4	91.2 ± 7.7	90.7 ± 8.1	90.4 ± 8.2	90.2 ± 7.9	90.1 ± 7.7	90.0 ± 7.7	90.0 ± 7.7
Heart Rate	Day One	67.1 ± 8.9	67.4 ± 8.6	67.8 ± 8.7	67.7 ± 8.5	67.9 ± 8.6	67.9 ± 8.7	68.0 ± 8.7	68.1 ± 8.7
Heart Rate	Day Two	67.4 ± 9.0	67.3 ± 8.9	67.4 ± 8.8	67.4 ± 8.6	67.7 ± 8.7	67.8 ± 8.8	68.3 ± 8.8	68.4 ± 8.8
Heart Rate	Day Three	66.4 ± 6.4	66.9 ± 6.7	67.1 ± 6.9	67.0 ± 6.8	67.0 ± 6.8	67.0 ± 6.8	67.0 ± 6.8	67.1 ± 6.8

Data are displayed as mean ± standard deviation. Partial pressure end-tidal values of carbon dioxide ($P_{ET}CO_2$).

Supplemental Table 4: Neurovascular coupling metrics derived during a "*Where's Waldo?*" paradigm in 60 individuals (30 females / 30 males) in the posterior cerebral artery (PCA) and middle cerebral artery (MCA). Numerous of individuals completed repeated assessments, which produced a total of 160 unique data time points. Data were produced from the number of trials completed which ranged from one to eight.

Variable	One	Two	Three	Four	Five	Six	Seven	Eight
PCA Baseline	33.8 ± 6.4	33.8 ± 6.2	33.8 ± 6.2	33.8 ± 6.2	33.8 ± 6.2	33.7 ± 6.3	33.7 ± 6.3	33.7 ± 6.3
PCA Peak	45.5 ± 8.5	44.7 ± 8.2	44.3 ± 8.1	44.1 ± 8.0	43.8 ± 7.9	43.6 ± 7.8	43.4 ± 7.7	43.3 ± 7.7
PCA Percent Increase	35.0 ± 11.1	32.4 ± 9.7	31.3 ± 9.0	30.6 ± 9.0	29.9 ± 8.7	29.6 ± 8.9	29.1 ± 8.6	28.9 ± 8.6
PCA AUC30	210.2 ± 87.5	208.2 ± 80.4	207.1 ± 76.2	205.6 ± 73.7	204.1 ± 71.2	204.7 ± 71.6	203.1 ± 69.3	202.6 ± 69.2
PCA Time To Peak	16.9 ± 7.2	17.3 ± 6.5	17.7 ± 6.2	17.6 ± 6.1	17.8 ± 5.9	17.6 ± 6.2	17.6 ± 6.1	18.0 ± 6.2
MCA Baseline	58.4 ± 9.9	58.4 ± 9.8	58.3 ± 10	58.2 ± 10.1	58.2 ± 10.1	58.0 ± 10.1	57.9 ± 10.1	57.9 ± 10.1
MCA Peak	67.9 ± 11.5	65.9 ± 10.8	65.0 ± 10.6	64.6 ± 10.7	64.2 ± 10.5	63.9 ± 10.5	63.6 ± 10.6	63.5 ± 10.6
MCA Percent Increase	16.7 ± 10.1	13.3 ± 7.1	11.8 ± 6.4	11.3 ± 6.1	10.7 ± 5.6	10.6 ± 5.6	10.2 ± 5.2	9.9 ± 5.1
MCA AUC30	97.3 ± 97.3	86.8 ± 72.8	81.0 ± 67.3	78.5 ± 66.6	77.2 ± 64.7	79.2 ± 65.1	76.6 ± 60.4	76.4 ± 60.6
MCA Time To Peak	13.1 ± 9.1	13.5 ± 8.7	13.9 ± 9.3	13.6 ± 9.2	13.9 ± 9.4	14.0 ± 9.4	14.9 ± 9.7	13.3 ± 9.3

Data are displayed as mean ± standard deviation. The outcome metrics of interest within the PCA and MCA included: baseline velocity (cm/s), peak velocity (cm/s), relative percent (%) increase in velocity from baseline to peak, total activation/area-under-the-curve during the first 30-seconds of task engagement (AUC30) (cm/s/30s), and time-to-peak velocity during task engagement (s).

Supplemental Table 5: Neurovascular coupling metrics derived during a "*Where's Waldo?*" paradigm in 11 individuals (3 females / 8 males) in the posterior cerebral artery (PCA) and middle cerebral artery (MCA) at seven time points across a single day. Data were produced from the number of trials completed which ranged from one to eight.

Variable	Time Point	One	Two	Three	Four	Five	Six	Seven	Eight
PCA Baseline	08:00	34.1 ± 7.0	33.6 ± 6.9	33.4 ± 7.3	33.5 ± 7.3	33.6 ± 7.3	33.5 ± 7.3	33.4 ± 7.3	33.5 ± 7.3
PCA Baseline	09:30	33.3 ± 6.7	33.4 ± 6.9	33.7 ± 6.8	33.7 ± 6.8	33.7 ± 6.7	33.5 ± 6.5	33.5 ± 6.5	33.4 ± 6.4
PCA Baseline	10:30	33.2 ± 5.5	33.4 ± 6.3	33.5 ± 6.6	33.6 ± 6.5	33.6 ± 6.6	33.7 ± 6.6	33.7 ± 6.6	33.7 ± 6.7
PCA Baseline	11:30	33.3 ± 5.7	33.6 ± 5.5	33.6 ± 5.6	33.7 ± 6.0	33.6 ± 5.9	33.5 ± 6.0	33.5 ± 5.9	33.6 ± 5.8
PCA Baseline	13:30	34.1 ± 6.7	33.9 ± 6.5	34.1 ± 6.3	34.2 ± 6.3	34.3 ± 6.4	34.3 ± 6.3	34.2 ± 6.4	34.1 ± 6.3
PCA Baseline	15:30	32.9 ± 6.5	33.0 ± 6.5	32.9 ± 6.3	32.9 ± 6.2	32.9 ± 6.2	32.9 ± 6.4	32.8 ± 6.3	32.8 ± 6.3
PCA Baseline	17:30	33.4 ± 6.0	32.6 ± 5.0	32.8 ± 5.2	32.8 ± 5.3	32.8 ± 5.3	32.6 ± 5.3	32.6 ± 5.4	32.6 ± 5.5
PCA Peak	08:00	45.4 ± 7.6	44.8 ± 7.6	44.3 ± 7.9	43.9 ± 7.7	43.6 ± 7.8	43.3 ± 7.6	43.3 ± 7.5	43.1 ± 7.5
PCA Peak	09:30	45.9 ± 9.8	44.8 ± 9.0	44.5 ± 8.6	44.8 ± 8.4	44.5 ± 8.7	44.2 ± 8.4	44.0 ± 7.9	43.6 ± 7.8
PCA Peak	10:30	45.2 ± 7.8	44.9 ± 8.2	44.3 ± 8.2	44.2 ± 8.3	43.9 ± 8.0	43.7 ± 7.8	43.6 ± 7.7	43.5 ± 7.6
PCA Peak	11:30	45.2 ± 8.2	45.1 ± 8.0	44.8 ± 7.8	44.4 ± 7.7	44.2 ± 7.3	44.1 ± 7.0	43.8 ± 6.9	43.7 ± 6.9
PCA Peak	13:30	45.7 ± 9.0	45.2 ± 8.4	45.0 ± 8.3	44.7 ± 8.1	44.4 ± 8.0	44.3 ± 7.8	44.0 ± 7.8	44.0 ± 7.8
PCA Peak	15:30	43.8 ± 8.8	43.1 ± 8.1	43.1 ± 7.7	42.8 ± 7.4	42.6 ± 7.5	42.4 ± 7.3	42.3 ± 7.6	42.2 ± 7.4
PCA Peak	17:30	44.6 ± 7.5	43.1 ± 6.8	42.9 ± 6.7	42.6 ± 6.6	42.5 ± 6.7	42.5 ± 6.7	42.1 ± 6.8	42.2 ± 6.8
PCA Percent Increase	08:00	34.2 ± 7.7	34.0 ± 7.9	33.7 ± 8.0	32.4 ± 8.0	30.9 ± 7.7	30.4 ± 8.1	30.7 ± 8.2	29.9 ± 8.3
PCA Percent Increase	09:30	38.0 ± 9.1	34.4 ± 7.9	32.5 ± 7.4	33.3 ± 8.2	32.4 ± 7.4	32.3 ± 7.6	31.8 ± 7.3	31.3 ± 6.8
PCA Percent Increase	10:30	36.0 ± 9.4	34.9 ± 8.5	32.6 ± 6.4	31.8 ± 6.0	31.0 ± 6.7	30.3 ± 6.8	30.0 ± 7.3	29.8 ± 7.4
PCA Percent Increase	11:30	35.8 ± 13.6	34.4 ± 9.0	33.6 ± 9.1	32.1 ± 9.6	32.0 ± 8.8	32.0 ± 8.5	31.4 ± 8.0	30.4 ± 7.8
PCA Percent Increase	13:30	34.4 ± 7.9	33.8 ± 6.8	32.1 ± 6.7	30.9 ± 7.4	30.0 ± 7.7	29.5 ± 7.8	29.1 ± 7.5	29.3 ± 8.0
PCA Percent Increase	15:30	33.7 ± 10.7	31.3 ± 11.6	31.7 ± 9.6	30.8 ± 9.1	30.0 ± 8.5	29.6 ± 8.7	29.4 ± 8.0	28.8 ± 7.6
PCA Percent Increase	17:30	33.8 ± 8.6	32.3 ± 6.5	31.3 ± 6.5	30.1 ± 7.1	29.9 ± 7.5	30.4 ± 7.8	29.6 ± 7.4	29.9 ± 6.9
PCA AUC30	08:00	211.6 ± 55.7	221.3 ± 60.0	225.9 ± 46.6	219.0 ± 45.6	215.8 ± 46.0	212.0 ± 42.1	213.1 ± 40.3	209.9 ± 40.4
PCA AUC30	09:30	224.1 ± 49.2	224.1 ± 64.2	216.0 ± 62.4	217.7 ± 58.0	216.7 ± 60.2	217.8 ± 61.0	216.3 ± 57.0	216.4 ± 55.0
PCA AUC30	10:30	240.9 ± 91.0	231.6 ± 65.9	217.0 ± 56.2	215.8 ± 53.0	211.2 ± 51.6	209.7 ± 52.3	208.0 ± 53.2	207.8 ± 51.2
PCA AUC30	11:30	214.0 ± 90.6	220.4 ± 69.3	221.9 ± 67.2	216.2 ± 63.6	217.6 ± 59.1	222.9 ± 53.4	224.2 ± 54.2	219.5 ± 56.7
PCA AUC30	13:30	207.6 ± 65.7	220.2 ± 52.0	215.0 ± 53.2	209.5 ± 54.0	207.8 ± 55.1	207.0 ± 56.4	207.8 ± 54.3	209 ± 55.1
PCA AUC30	15:30	196.9 ± 69.4	200.2 ± 73.6	203.4 ± 61.8	200.6 ± 55.3	199.0 ± 52.6	198.7 ± 52.4	202.2 ± 52.5	200.1 ± 50.9
PCA AUC30	17:30	183.6 ± 70.9	210 ± 62.5	206.8 ± 59.2	204.2 ± 63.0	202.9 ± 62.3	207.3 ± 61.2	204.1 ± 57.1	203.3 ± 56.6
PCA Time to Peak	08:00	19.8 ± 6.6	17.9 ± 6.4	17.5 ± 5.7	16.0 ± 5.0	17.2 ± 4.4	16.7 ± 4.8	16.9 ± 4.6	18.5 ± 6.5
PCA Time to Peak	09:30	19.5 ± 6.6	18.8 ± 6.3	18.2 ± 4.1	19.9 ± 5.6	19.5 ± 5.2	19.9 ± 6.1	19.2 ± 5.6	19.2 ± 5.3
PCA Time to Peak	10:30	17.9 ± 6.5	18.6 ± 5.0	17.5 ± 3.5	16.6 ± 2.8	17.3 ± 4.6	17.7 ± 3.4	17.6 ± 4.8	19.0 ± 4.1
PCA Time to Peak	11:30	16.7 ± 7.1	18.7 ± 5.7	16.1 ± 4.8	17.8 ± 4.5	18.2 ± 5.6	16.7 ± 6.3	19.4 ± 6.2	19.3 ± 5.9
PCA Time to Peak	13:30	18.7 ± 6.5	18.8 ± 5.6	16.8 ± 4.2	16.9 ± 4.1	16.6 ± 4.0	16.8 ± 4.0	18.0 ± 5.0	18.2 ± 4.7
PCA Time to Peak	15:30	14.6 ± 4.8	17.9 ± 5.8	18.8 ± 5.1	19.2 ± 5.6	18.3 ± 5.1	17.6 ± 5.4	17.6 ± 5.1	17.6 ± 5.5
PCA Time to Peak	17:30	16.0 ± 7.7	18.9 ± 6.9	19.6 ± 5.6	19.9 ± 6.0	19.8 ± 6.0	20.1 ± 6.5	19.7 ± 6.1	18.8 ± 6.2

MCA Baseline	08:00	61.5 ± 9.9	60.4 ± 9.3	60 ± 10.1	60.1 ± 10.2	60.1 ± 10.4	59.9 ± 10.5	59.9 ± 10.5	59.9 ± 10.5
MCA Baseline	09:30	59.1 ± 9.6	59.6 ± 9.6	59.9 ± 9.3	59.8 ± 9.2	59.6 ± 8.9	59.4 ± 8.9	59.3 ± 8.9	59.3 ± 8.9
MCA Baseline	10:30	59.3 ± 6.5	59.5 ± 7.4	60.1 ± 8.0	60.1 ± 8.2	60.1 ± 8.4	60.1 ± 8.6	60.2 ± 8.6	60.2 ± 8.7
MCA Baseline	11:30	59.2 ± 8.6	59.6 ± 8.8	59.8 ± 9.7	59.8 ± 10.2	59.6 ± 10.1	59.6 ± 10.0	59.5 ± 9.8	59.4 ± 9.5
MCA Baseline	13:30	61.7 ± 9.9	61.6 ± 9.0	61.9 ± 9.3	62.3 ± 9.3	62.6 ± 9.8	62.5 ± 9.9	62.2 ± 9.7	62.1 ± 9.8
MCA Baseline	15:30	60 ± 9.7	59.0 ± 8.8	58.9 ± 8.3	58.8 ± 8.3	59.0 ± 8.3	59.0 ± 8.3	58.8 ± 8.2	59.0 ± 8.1
MCA Baseline	17:30	61.8 ± 8.1	59.9 ± 7.2	59.9 ± 6.7	59.8 ± 7.3	59.6 ± 7.6	59.2 ± 7.6	59.0 ± 7.8	58.9 ± 7.9
MCA Peak	08:00	69.2 ± 9.9	67.1 ± 9.5	66.2 ± 9.8	65.4 ± 9.9	65.2 ± 9.8	65.1 ± 10	64.9 ± 10	64.6 ± 10.1
MCA Peak	09:30	68.1 ± 9.8	66.4 ± 9.5	66.0 ± 9.0	65.7 ± 8.7	65.1 ± 8.9	64.7 ± 8.8	64.4 ± 8.9	64.0 ± 8.7
MCA Peak	10:30	68.7 ± 8.5	67.6 ± 8.6	66.5 ± 8.4	65.8 ± 8.7	65.2 ± 8.2	65.0 ± 7.9	64.8 ± 8.1	64.8 ± 8.2
MCA Peak	11:30	67.6 ± 10.6	66.8 ± 10.7	65.5 ± 10.9	65.1 ± 10.4	64.9 ± 9.9	64.9 ± 9.5	64.8 ± 9.5	64.7 ± 9.5
MCA Peak	13:30	70.8 ± 10.7	68.6 ± 9.9	68.1 ± 10.1	68.0 ± 10.1	67.5 ± 9.9	67.3 ± 9.7	67.1 ± 9.8	67.0 ± 9.8
MCA Peak	15:30	66.9 ± 8.9	64.7 ± 7.9	64.4 ± 7.4	64.3 ± 7.2	63.9 ± 7.2	63.9 ± 7.2	63.6 ± 7.3	63.5 ± 7.2
MCA Peak	17:30	68.7 ± 9.6	66.1 ± 7.9	65.4 ± 7.5	65.1 ± 8.0	64.8 ± 7.6	64.6 ± 7.8	64.2 ± 8.4	64.0 ± 8.8
MCA Percent Increase	08:00	13.0 ± 5.1	11.4 ± 4.6	10.8 ± 5.1	9.2 ± 4.3	8.9 ± 4.8	9.1 ± 4.3	8.9 ± 4.2	8.3 ± 4.3
MCA Percent Increase	09:30	16.1 ± 7.5	11.9 ± 6.3	10.7 ± 5.1	10.4 ± 5.8	9.5 ± 5.0	9.2 ± 4.9	8.9 ± 4.5	8.3 ± 4.8
MCA Percent Increase	10:30	16.0 ± 7.5	13.8 ± 4.2	10.7 ± 3.1	9.5 ± 2.9	8.7 ± 3.4	8.5 ± 4.3	8.0 ± 4.0	7.9 ± 4.0
MCA Percent Increase	11:30	14.2 ± 8.8	12.0 ± 6.1	9.6 ± 4.3	9.1 ± 5.1	9.1 ± 4.6	9.4 ± 5.3	9.4 ± 5.2	9.2 ± 4.7
MCA Percent Increase	13:30	15.2 ± 9.4	11.8 ± 7.1	10.1 ± 5.5	9.3 ± 5.1	8.1 ± 3.5	8.1 ± 3.5	8.0 ± 3.1	8.1 ± 3.1
MCA Percent Increase	15:30	12.5 ± 9.4	10.2 ± 6.8	10.0 ± 6.8	9.9 ± 7.0	8.9 ± 6.3	8.9 ± 6.0	8.5 ± 5.4	8.1 ± 5.3
MCA Percent Increase	17:30	11.3 ± 6.6	10.6 ± 4.1	9.1 ± 2.8	8.8 ± 2.4	8.8 ± 3.1	9.3 ± 3.1	8.9 ± 3.1	8.6 ± 2.8
MCA AUC30	08:00	63.7 ± 62.2	78.4 ± 74.5	81.4 ± 65.5	69.4 ± 65.1	67.0 ± 64.4	70.2 ± 56.2	69.6 ± 51.0	65.8 ± 48.9
MCA AUC30	09:30	103.9 ± 59.8	93.7 ± 78.1	72.8 ± 58.4	64.8 ± 60.2	59.0 ± 62.4	60.3 ± 60.1	57.7 ± 51.3	55.0 ± 49.1
MCA AUC30	10:30	129.6 ± 115.3	114.9 ± 65.0	80.4 ± 50.5	74.3 ± 46.4	69.8 ± 50.5	66.2 ± 53.9	63.8 ± 51.9	64.3 ± 47.5
MCA AUC30	11:30	95.3 ± 95.9	74.2 ± 59.0	63.2 ± 58.3	58.1 ± 59.4	57.6 ± 57.8	63.6 ± 57.5	67.5 ± 59.0	69.5 ± 64.4
MCA AUC30	13:30	63.1 ± 83.0	66.4 ± 60.3	59.3 ± 45.9	55.2 ± 42.3	48.2 ± 34.8	50.8 ± 38.9	54.1 ± 42.5	55.2 ± 42.2
MCA AUC30	15:30	67.6 ± 79.3	73.6 ± 71.9	68.4 ± 62.0	61.9 ± 58.5	55.8 ± 50.8	56.4 ± 51.1	55.9 ± 53.0	50.4 ± 47.7
MCA AUC30	17:30	46.1 ± 76.2	54.7 ± 37.1	43.7 ± 40.2	42.5 ± 46.0	45.7 ± 46.1	56.2 ± 45.5	54.1 ± 44.7	54.9 ± 46.0
MCA Time to Peak	08:00	13.8 ± 8.5	12.0 ± 7.7	14.1 ± 8.2	11.2 ± 8.0	9.7 ± 6.2	12.6 ± 7.5	15.8 ± 7.6	10.9 ± 7.7
MCA Time to Peak	09:30	15.6 ± 11.0	13.1 ± 8.6	11.3 ± 10.4	12.2 ± 10.0	12.5 ± 8.3	13.7 ± 9.7	11.4 ± 8.8	9.7 ± 7.1
MCA Time to Peak	10:30	12.3 ± 11.0	16.2 ± 8.8	14.4 ± 9.1	17.4 ± 8.2	18.6 ± 8.9	14.7 ± 9.9	15.2 ± 9.5	16.7 ± 9.7
MCA Time to Peak	11:30	11.8 ± 8.1	15.8 ± 9.2	9.6 ± 8.5	10.6 ± 7.9	9.4 ± 8.2	8.3 ± 6.9	10.5 ± 9.5	8.8 ± 7.1
MCA Time to Peak	13:30	12.0 ± 11.3	14.9 ± 10.2	12.8 ± 10.1	9.6 ± 7.0	11.8 ± 8.4	10.6 ± 9.4	11.1 ± 8.7	8.7 ± 7.0
MCA Time to Peak	15:30	12.4 ± 8.2	12.9 ± 7.7	14.3 ± 7.6	15.2 ± 7.7	12.9 ± 7.9	11.6 ± 7.5	14.0 ± 8.6	12.9 ± 9.4
MCA Time to Peak	17:30	10.6 ± 8.3	15.2 ± 8.3	19.5 ± 10.0	13.8 ± 11.1	16.1 ± 11.3	16.9 ± 9.9	17.1 ± 10.0	15.2 ± 8.8

Data are displayed as mean ± standard deviation. The outcome metrics of interest within the PCA and MCA included: baseline velocity (cm/s), peak velocity (cm/s), relative percent (%) increase in velocity from baseline to peak, total activation/area-under-the-curve during the first 30-seconds of task engagement (AUC30) (cm/s/30s), and time-to-peak velocity during task engagement (s).

Supplemental Table 6: Neurovascular coupling metrics derived during a "*Where's Waldo?*" paradigm in 17 individuals (3 females / 14 males) in the posterior cerebral artery (PCA) and middle cerebral artery (MCA) at three time points on separate days. Data were produced from the number of trials completed which ranged from one to eight.

Variable	Time Point	One	Two	Three	Four	Five	Six	Seven	Eight
PCA Baseline	Day One	33.2 ± 4.9	33.1 ± 4.9	33.1 ± 5.3	33.1 ± 5.3	33.1 ± 5.3	33.0 ± 5.3	33.0 ± 5.2	33.0 ± 5.2
PCA Baseline	Day Two	33.2 ± 7.2	32.8 ± 6.7	32.4 ± 6.7	32.2 ± 6.7	32.2 ± 6.7	32.2 ± 6.8	32.2 ± 6.6	32.2 ± 6.7
PCA Baseline	Day Three	34.5 ± 7.7	34.4 ± 7.3	34.5 ± 7.2	34.4 ± 7.2	34.4 ± 7.3	34.3 ± 7.5	34.4 ± 7.7	34.5 ± 7.8
PCA Peak	Day One	44.7 ± 7.0	43.7 ± 6.4	43.2 ± 6.8	43 ± 6.6	42.7 ± 6.6	42.5 ± 6.6	42.4 ± 6.4	42.3 ± 6.5
PCA Peak	Day Two	43.2 ± 8.3	42.4 ± 8.4	41.9 ± 8.1	41.8 ± 8.0	41.5 ± 7.8	41.4 ± 7.7	41.2 ± 7.6	41.1 ± 7.5
PCA Peak	Day Three	46.8 ± 9.8	45.8 ± 9.8	45.1 ± 10	44.8 ± 9.9	44.5 ± 9.9	44.3 ± 9.7	44.1 ± 9.7	44.1 ± 9.6
PCA Percent Increase	Day One	34.6 ± 9.9	32.1 ± 9.6	30.9 ± 9.3	30.4 ± 8.5	29.3 ± 8.3	28.9 ± 8.8	28.8 ± 8.5	28.5 ± 8.6
PCA Percent Increase	Day Two	30.8 ± 9.4	29.3 ± 8.6	29.7 ± 7.8	30.4 ± 7.9	29.7 ± 7.4	29.3 ± 7.8	28.6 ± 7.3	28.5 ± 7.5
PCA Percent Increase	Day Three	36.4 ± 9.6	32.9 ± 6.6	30.8 ± 6.4	29.7 ± 6.1	29.3 ± 6.8	29.1 ± 7.5	28.5 ± 7.9	28.0 ± 8.2
PCA AUC30	Day One	209.9 ± 73.3	202.4 ± 76.4	203.8 ± 68.4	203.7 ± 63.6	201.2 ± 65.3	199.8 ± 65.3	199.8 ± 64.5	199.6 ± 64.5
PCA AUC30	Day Two	169.8 ± 80.2	171.3 ± 71.9	187.2 ± 58.8	194.0 ± 55.8	196.6 ± 49.6	195.0 ± 49.8	194.9 ± 50.2	195.8 ± 51.1
PCA AUC30	Day Three	222.7 ± 62.9	215.4 ± 65.9	209.3 ± 77.0	207.8 ± 76.9	206.9 ± 74.6	204.5 ± 72.4	200.4 ± 67.1	198.1 ± 67.2
PCA Time To Peak	Day One	16.8 ± 7.0	15.8 ± 7.2	18.7 ± 7.5	17.1 ± 7.5	16.3 ± 6.8	17.1 ± 7.6	16.2 ± 6.8	19.6 ± 7.1
PCA Time To Peak	Day Two	15.4 ± 7.4	16.6 ± 6.5	18.6 ± 7.0	18.1 ± 6.6	19.1 ± 5.1	17.6 ± 6.5	15.0 ± 5.5	16.2 ± 6.5
PCA Time To Peak	Day Three	18.6 ± 7.6	18.2 ± 7.7	17.3 ± 8.5	18.2 ± 8.8	19.4 ± 7.7	19.0 ± 7.6	18.3 ± 7.1	16.8 ± 7.5
MCA Baseline	Day One	60.5 ± 8.2	59.3 ± 7.4	59.0 ± 7.7	58.9 ± 8.2	58.9 ± 8.3	58.7 ± 8.3	58.7 ± 8.1	58.6 ± 8.0
MCA Baseline	Day Two	60.0 ± 9.7	59.0 ± 8.8	58.9 ± 8.3	58.8 ± 8.3	59.0 ± 8.3	59.0 ± 8.3	58.8 ± 8.2	59.0 ± 8.1
MCA Baseline	Day Three	61.8 ± 8.1	59.9 ± 7.2	59.9 ± 6.7	59.8 ± 7.3	59.6 ± 7.6	59.2 ± 7.6	59.0 ± 7.8	58.9 ± 7.9
MCA Peak	Day One	69.2 ± 9.9	67.1 ± 9.5	66.2 ± 9.8	65.4 ± 9.9	65.2 ± 9.8	65.1 ± 10.0	64.9 ± 10.0	64.6 ± 10.1
MCA Peak	Day Two	68.1 ± 9.8	66.4 ± 9.5	66.0 ± 9.0	65.7 ± 8.7	65.1 ± 8.9	64.7 ± 8.8	64.4 ± 8.9	64.0 ± 8.7
MCA Peak	Day Three	68.7 ± 9.6	66.1 ± 7.9	65.4 ± 7.5	65.1 ± 8.0	64.8 ± 7.6	64.6 ± 7.8	64.2 ± 8.4	64.0 ± 8.8
MCA Percent Increase	Day One	13.0 ± 5.1	11.4 ± 4.6	10.8 ± 5.1	9.2 ± 4.3	8.9 ± 4.8	9.1 ± 4.3	8.9 ± 4.2	8.3 ± 4.3
MCA Percent Increase	Day Two	12.5 ± 9.4	10.2 ± 6.8	10.0 ± 6.8	9.9 ± 7.0	8.9 ± 6.3	8.9 ± 6.0	8.5 ± 5.4	8.1 ± 5.3
MCA Percent Increase	Day Three	11.3 ± 6.6	10.6 ± 4.1	9.1 ± 2.8	8.8 ± 2.4	8.8 ± 3.1	9.3 ± 3.1	8.9 ± 3.1	8.6 ± 2.8
MCA AUC30	Day One	63.7 ± 62.2	78.4 ± 74.5	81.4 ± 65.5	69.4 ± 65.1	67.0 ± 64.4	70.2 ± 56.2	69.6 ± 51.0	65.8 ± 48.9
MCA AUC30	Day Two	67.6 ± 79.3	73.6 ± 71.9	68.4 ± 62.0	61.9 ± 58.5	55.8 ± 50.8	56.4 ± 51.1	55.9 ± 53.0	50.4 ± 47.7
MCA AUC30	Day Three	46.1 ± 76.2	54.7 ± 37.1	43.7 ± 40.2	42.5 ± 46.0	45.7 ± 46.1	56.2 ± 45.5	54.1 ± 44.7	54.9 ± 46.0
MCA Time To Peak	Day One	13.8 ± 8.5	12.0 ± 7.7	14.1 ± 8.2	11.2 ± 8.0	9.7 ± 6.2	12.6 ± 7.5	15.8 ± 7.6	10.9 ± 7.7
MCA Time To Peak	Day Two	12.4 ± 8.2	12.9 ± 7.7	14.3 ± 7.6	15.2 ± 7.7	12.9 ± 7.9	11.6 ± 7.5	14.0 ± 8.6	12.9 ± 9.4
MCA Time To Peak	Day Three	10.6 ± 8.3	15.2 ± 8.3	19.5 ± 10.0	13.8 ± 11.1	16.1 ± 11.3	16.9 ± 9.9	17.1 ± 10.0	15.2 ± 8.8

Data are displayed as mean ± standard deviation. The outcome metrics of interest within the PCA and MCA included: baseline velocity (cm/s), peak velocity (cm/s), relative percent (%) increase in velocity from baseline to peak, total activation/area-under-the-curve during the first 30-seconds of task engagement (AUC30) (cm/s/30s), and time-to-peak velocity during task engagement (s).
