# Supplementary material for: Neurovascular coupling on trial: How the number of trials completed impacts the accuracy and precision of temporally derived neurovascular coupling estimates
Source: J Cereb Blood Flow Metab. 2022 Feb 25;42(8):1478–92. doi: 10.1177/0271678X221084400 (PMC9274868; doi:10.1177/0271678X221084400)
Supplement: sj-pdf-5-jcb-10.1177_0271678X221084400 - Supplemental material for Neurovascular coupling on trial: How the number of trials completed impacts the accuracy and precision of temporally derived neurovascular coupling estimates [file sj-pdf-5-jcb-10.1177_0271678X221084400.pdf]

Supplemental Figure 1: Bland-Altman plots with 95% limits of agreement demonstrating the *concurrent validity* of neurovascular coupling metrics within the middle cerebral artery (MCA) derived from one to seven trials compared to the "*reference-standard*" eight trials. A total of 160 time points were drawn from 60 participants (30 females / 30 males). It is important to note the increasing trials represent the average from the given numbers of trials completed, which would contain the previous trial(s). It should be highlighted the time-to-peak measures displayed wide variance across all trials and therefore this metric should be interpreted with caution. The outcome metrics of interest within the middle cerebral artery (MCA) included: baseline MCA velocity (cm/s), peak MCA velocity (cm/s), relative percent (%) increase in MCA velocity from baseline to peak, MCA total activation/area-under-the-curve during the first 30-seconds of task engagement (AUC30) (cm/s/30s), and time-to-peak MCA velocity during task engagement (s).

Supplemental Figure 2: Intraclass correlation coefficients (ICC), adjusted coefficient of determination (r^2) values, and coefficient of variation (COV) metrics demonstrating the *concurrent validity* of neurovascular coupling metrics within the middle cerebral artery (MCA) derived from one to seven trials compared to the "*reference-standard*" eight trials. It is important to note the increasing trials represent the average from the given numbers of trials completed, which would contain the previous trial(s). Thresholds for the ICC were set at: <0.50 (poor; red), 0.50 – 0.75 (moderate; orange), 0.75 – 0.90 (good; yellow), and >0.90 (excellent; green). Thresholds for the adjusted r^2 metrics were set at: <0.10 (negligible; red), 0.10 – 0.30 (small; orange), 0.30 – 0.50 (moderate; yellow), 0.50 – 0.80 (large; blue), and 0.80 – 1.00 (very large; green). Thresholds for the CoV were: >20% (unacceptable; red), 10 – 20% (acceptable: orange), 5 – 10% (good; yellow), and <5% (excellent; green). The outcome metrics of interest within the middle cerebral artery

(MCA) included: baseline MCA velocity (cm/s), peak MCA velocity (cm/s), relative percent (%) increase in MCA velocity from baseline to peak, MCA total activation/area-under-the-curve during the first 30-seconds of task engagement (AUC30) (cm/s/30s), and time-to-peak MCA velocity during task engagement (s).

Supplemental Figure 3: Intraclass correlation coefficients (ICC) and coefficient of variation (COV) metrics demonstrating the *within-day reliability* of neurovascular coupling metrics within the middle cerebral artery (MCA) derived from one to eight trials. It is important to note the increasing trials represent the average from the given numbers of trials completed, which would contain the previous trial(s). Thresholds for the ICC were set at: <0.50 (poor; red), 0.50 – 0.75 (moderate; orange), 0.75 – 0.90 (good; yellow), and >0.90 (excellent; green). Thresholds for the CoV were: >20% (unacceptable; red), 10 – 20% (acceptable: orange), 5 – 10% (good; yellow), and <5% (excellent; green). The outcome metrics of interest within the middle cerebral artery (MCA) included: baseline MCA velocity (cm/s), peak MCA velocity (cm/s), relative percent (%) increase in MCA velocity from baseline to peak, MCA total activation/area-under-the-curve during the first 30-seconds of task engagement (AUC30) (cm/s/30s), and time-to-peak MCA velocity during task engagement (s).

Supplemental Figure 4: Intraclass correlation coefficients (ICC) and coefficient of variation (COV) metrics demonstrating the *between-day reliability* of neurovascular coupling metrics within the middle cerebral artery (MCA) derived from one to eight trials. It is important to note the increasing trials represent the average from the given numbers of trials completed, which would contain the previous trial(s). Thresholds for the ICC were set at: <0.50 (poor; red), 0.50 – 0.75

(moderate; orange), 0.75 – 0.90 (good; yellow), and >0.90 (excellent; green). Thresholds for the CoV were: >20% (unacceptable; red), 10 – 20% (acceptable: orange), 5 – 10% (good; yellow), and <5% (excellent; green). The outcome metrics of interest within the middle cerebral artery (MCA) included: baseline MCA velocity (cm/s), peak MCA velocity (cm/s), relative percent (%) increase in MCA velocity from baseline to peak, MCA total activation/area-under-the-curve during the first 30-seconds of task engagement (AUC30) (cm/s/30s), and time-to-peak MCA velocity during task engagement (s).
